# Fine-tuning Bacterial Cyclic di-AMP Production for Durable Antitumor Effects Through the Activation of the STING Pathway

**DOI:** 10.34133/research.0102

**Published:** 2023-03-30

**Authors:** Yu Jiang, Xiyuan Li, Fenghui Qian, Bingbing Sun, Xiyuan Wang, Yan Zhang, Deqiang Zhang, Meiyu Geng, Zuoquan Xie, Sheng Yang

**Affiliations:** ^1^ Shanghai Research and Development Center of Industrial Biotechnology, Shanghai, China.; ^2^ State Key Laboratory of Drug Research, Shanghai Institute of Materia Medica, Chinese Academy of Sciences, Shanghai, China.; ^3^ School of Life Science and Technology, ShanghaiTech University, Shanghai, China.; ^4^ University of Chinese Academy of Sciences, Beijing, China.; ^5^ Key Laboratory of Synthetic Biology, CAS Center for Excellence in Molecular Plant Sciences, Chinese Academy of Sciences, Shanghai, China.; ^6^ Huzhou Research Center of Industrial Biotechnology, Shanghai Institutes for Biological Sciences, Chinese Academy of Sciences, Huzhou, China; ^7^ Shanghai Taoyusheng Biotechology Co. Ltd. Shanghai, China.

## Abstract

The stimulator of interferon genes (STING) protein is an important and promising innate immune target for tumor therapy. However, the instability of the agonists of STING and their tendency to cause systemic immune activation is a hurdle. The STING activator, cyclic di-adenosine monophosphate (CDA), produced by the modified *Escherichia coli* Nissle 1917, shows high antitumor activity and effectively reduces the systemic effects of the “off-target” caused by the activation of the STING pathway. In this study, we used synthetic biological approaches to optimize the translation levels of the diadenylate cyclase that catalyzes CDA synthesis in vitro. We developed 2 engineered strains, CIBT4523 and CIBT4712, for producing high levels of CDA while keeping their concentrations within a range that did not compromise the growth. Although CIBT4712 exhibited stronger induction of the STING pathway corresponding to in vitro CDA levels, it had lower antitumor activity than CIBT4523 in an allograft tumor model, which might be related to the stability of the surviving bacteria in the tumor tissue. CIBT4523 exhibited complete tumor regression, prolonged survival of mice, and rejection of rechallenged tumors, thus, offering new possibilities for more effective tumor therapy. We showed that the appropriate production of CDA in engineered bacterial strains is essential for balancing antitumor efficacy and self-toxicity.

## Introduction

The stimulator of interferon genes (STING) protein is a central mediator of the activation of innate immunity in the cytosol in response to double-stranded DNA, which is expressed in various immune cells, such as macrophages, dendritic cells (DCs), and T cells, as well as endothelial and epithelial cells [1]. STING can be activated by eukaryote-derived cyclic guanosine monophosphate-adenosine monophosphate (cyclic GMP-AMP) [2,3] and bacteria-derived cyclic di-AMP (CDA) [4] or cyclic di-GMP [5]. Then, it recruits and activates the TANK-binding kinase 1 (TBK1)/interferon (IFN) regulatory factor 3 (IRF3) and nuclear factor κB (NF-κB) pathways to initiate the innate immune response [1]. When dendritic cells phagocytose tumor cells, the STING pathway is triggered by tumor cell-derived DNA, which then induces type I IFN and promotes antigen presentation and activation of antigen-specific T cells for eliminating tumors [6]. Thus, STING strongly influences immune surveillance, and STING activation is a novel antitumor strategy.

There are 2 known classes of STING agonists, including cyclic dinucleotides (CDNs) and non-CDNs [7]. CDNs are mainly modified by natural ligands, while they are unstable and difficulty in production [8]. Non-CDNs include small-molecule agonists, such as diABZI (linked amidobenzimidazole compound 3) [9], MSA-2 (benzothiopheneoxobutanoic acid) [10], and SR-717 [11], but they lack cell selectivity and might cause cytokine storms. Therefore, the development of new therapeutic strategies to selectively activate STING in certain immune cells has become a hotspot of current research. Such studies are important for the safe and effective application of STING agonists in antitumor therapy.

Bacterium-based cancer therapies were first performed during the identification and testing of the Coley toxin [12] in 1891 when live streptococcal bacteria were injected into the tumors of the patients. These early observations and numerous subsequent studies showed that various bacteria have the inherent ability to selectively colonize tumors, primarily in the hypoxic tumor core, which might even lead to tumor regression [13]. The only microbe-based cancer therapy in clinical practice is the Bacillus Calmette–Guerin (BCG) therapy, which has been applied for treating bladder cancer for over 30 years, thus confirming its clinical accessibility and effectiveness [14]. With the development of tumor immunotherapy, major pharmaceutical companies and start-ups around the world have laid out an oncolytic bacteria pipeline [15]; specifically, Bacillus Calmette–Guerin combined with immune checkpoint antibodies for bladder cancer has entered phase III clinical trials, conducted by AstraZeneca, Roche, etc. (NCT03528694, NCT03799835, NCT03149574, NCT03711032, and NCT03519256).

The recent advancements in synthetic biology have allowed researchers to engineer naturally tumor-targeting bacteria for controlling the expression of metabolites, cytokines, and other molecules that participate in cellular immune responses [16]. Bacteria have also been engineered to be mediated by multifunctional nanoparticles combined with focused ultrasonic ablation surgery to improve the effectiveness of tumor treatment [17]. The engineering of bacterial cells to produce CDNs for activating STING, along with the natural immune-engaging components present in bacterial cells, can drive durable antitumor activity, even in “cold” tumors [18]. This technique can also reduce systemic effects by activating STING in “off-target” cell types [15]. Additionally, the continuous supply of CDNs by live bacterial cells helps to overcome the problems related to the instability of CDNs in vivo [19]. *Escherichia coli* Nissle 1917 (EcN) is a commercially available tool strain used for developing various therapeutic products [20]. However, no study has determined the effect of the protein expression system on the production of CDA. Additionally, the optimum level of CDA production also remains unknown, considering that excessive CDA is toxic to the CDA-producing bacteria [21], which might affect the large-scale manufacturing of CDA.

In this study, we determined the effect of modifying diadenylate cyclase encoded by the *dacA* expression system on the production of CDA in EcN. We obtained 2 engineered stains (CIBT4523 and CIBT4712) and systematically evaluated their activity at the cellular level and in the allograft tumor models of mice. We also showed that the stability of the engineered strain is important for the antitumor effect in vivo, which might induce a stronger antitumor immune response in the tumor microenvironment and a higher increase in the immune-activating cytokines found in the blood. Our findings provided important information for the clinical application of CIBT4523 in the future.

## Results

### Comparison of 3 CDNs produced by recombinant EcN

We selected 3 types of *E. coli* codon-optimized enzymes, including diadenylate cyclase, nucleotide transferase, and diguanylate cyclase. We expressed them using the plasmid pMW119K under the control of the tetracycline-inducible promoter to produce 3 CDNs, including CDA, 3′3′-cyclic GMP-AMP, and cyclic di-GMP. Recombinant EcN containing diadenylate cyclase produced about 10 μM CDA without induction, while the production of CDA was considerably lower under anhydrotetracycline (ATC) induction, which might be related to the cytotoxicity of the overproduction of CDA. The products such as 3′3′-cyclic GMP-AMP and cyclic di-GMP were not detected without induction, while their concentrations were considerably lower than that of CDA under ATC induction. Therefore, the recombinant EcN that produced CDA was selected for further investigation (Table S1).

### The toxicity regulation of *dacA* expression in EcN

**Fig. 1.**
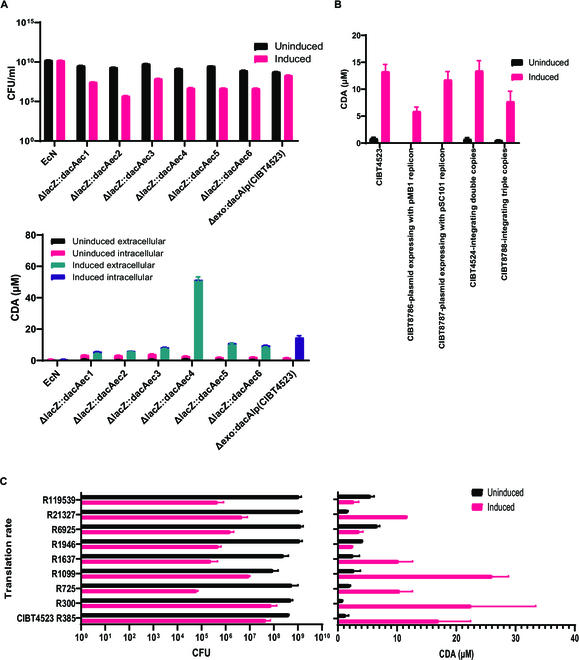
The regulation of toxicity during cyclic di-adenosine monophosphate (CDA) production in recombinant *Escherichia coli* Nissle 1917 (EcN). (A) The intracellular and extracellular CDA produced by recombinant EcN integrated with *E. coli* or *Lactobacillus* codon-optimized *dacA*, and the effect of the accumulation of CDA on growth, as indicated by the colony-forming unit (CFU) per milliliter in host EcN. (B) Optimizing the dosage of *dacA* for the production of CDA. The *Lactobacillus* codon-optimized *dacA* was expressed with high or low copy number plasmid (pMB1 or pSC101 replicon), or it was integrated into the genome with relative copy numbers. (C) The production of CDA and the effect of the ribosome binding site (RBS) sequence of *dacA* on growth, with the intensity value ranging from R300 to R119539, were determined using the Salislab online software.

The production of CDA via the expression of *dacA* in the plasmid was unstable (data not shown), which might be caused by the unstable expression of the gene in the plasmid; thus, *dacA* was integrated into the EcN genome. To better regulate the microbial agents for tumor treatment in vivo, a recombinant EcN in which *dapA* and *thyA* were knocked out was used for genome editing (CIBT4503); this double-deficient strain was unable to grow in tumors [15], which prevented its unlimited proliferation and increased toxicity in the body. Six positive transformants (lacZ: dacAec1 to dacAec6) died rapidly after ATC induction (Fig. 1A). Upon sequencing, we found that the induced strains all produced different mutations, deletions, or insertions in the open reading frame of *dacA* (Table S2). The strains with 3 types of mutations in the *dacA* gene were cultured for induction again, and no CDA or mutations were detected (data not shown). However, extracellular CDA production (up to 50.2 μM) occurred after induction among those transformants without *dacA* mutation, while the intracellular CDA concentration was generally low (1 to 3 μM) (Fig. 1A). These findings suggested that the cells rapidly lysed and released CDA because of the toxic effects of induction.

Next, we determined whether toxicity could be reduced by fine-tuning the degree of induction. For this, we used 0 to 200 ng/ml of ATC, induced for 1 to 5 h, and then performed lethality statistics. By reducing the induction time to 1 h, the colony-forming unit (CFU) was reduced by 10^2^ to 10^3^, which indicated that over 99% of the strains died. When the induction was increased to 3 to 5 h, the CFU increased again, which was probably because of the growth advantage of the mutant strain. The change in the induction dose did not reduce the lethality of the bacterial species (Table S3). These results suggested that reducing toxicity by only controlling the induction time and the inducer dose is difficult.

To find a stable strain with low-expression toxicity, we integrated the *Lactobacillus* codon-optimized *dacA* gene into CIBT4503 to form CIBT4523. After induction, cell death and *dacA* mutation were not detected. Approximately 14.3 μM intracellular CDA was produced by CIBT4523 (Fig. 1A). Therefore, the regulation of translation decreased the toxicity related to the expression of *dacA*.

### Optimization of CDA production with different gene dosages and ribosome binding sites

We first optimized the induction time and inducer dose of CIBT4523 for subsequent comparisons among different strains. The CIBT4523 strain exhibited the highest production of CDA when induced by 200 ng/ml of ATC for 4 h (Table S4), while no marked difference among the different concentrations of ATC was detected after 4.5 h of induction (Table S5). Therefore, the follow-up fixed induction time was 4.5 h, and the dosage of ATC used was 200 ng/ml.

Then, we tested the production of CDA by regulating the expression of *dacA* under different gene dosages. When *dacA* was expressed under the high-copy plasmid containing the replicon pMB1, the CFU decreased by 4 orders of magnitude after plate culture induction (Table S6). In contrast, the *dacA* expressed using a low-copy plasmid containing the replicon pSC101 did not show lethality. Additionally, the strains that integrated 2 copies of *dacA* in their genome had negligible changes in growth, while 3 integrated copies of *dacA* reduced the CFU. However, the regulation of these gene dosages did not enhance the production of CDA compared to the level of CDA in CIBT4523 (Fig. 1B). Thus, manipulating gene dosages did not increase the production of CDA.

Next, we determined whether CDA output could be increased by optimizing the ribosome binding site (RBS). Nine groups of RBS sequences with intensity values ranging from R300 to R119539, obtained from the Salis Lab online software (Table S7), were selected for testing. Four highly lethal strains (R1946, R6925, R21367, and R119539) were selected for sequencing. These colonies had various mutations (7 of 8) in *dacA*, *tetR*, and Ptet (Table S8), and their CDA production was also reduced from 1 to 5 μM. Among the remaining strains that did not affect growth, a slightly higher concentration of CDA was produced by the only 2 strains R300 and R1099 (Fig. 1C).

### Optimization of CDA production through the modification of the translation initiation region

**Fig. 2.**
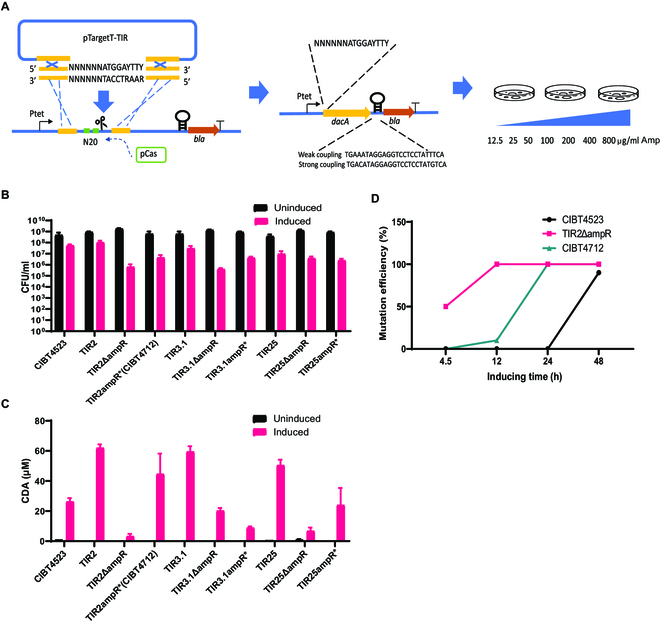
The translation initiation region (TIR) of *dacA* was optimized for the production of CDA. (A) A schematic representation of the principle for TIR selection by fusing an ampicillin-resistant gene as a reporter system to *dacA*. (B) The effect of the accumulation of CDA as indicated by the CFU per milliliter on the growth of host EcN of TIR-selected strains. (C) The production of CDA by TIR-selected strains. (D) The mutation rate–time curves of CIBT4523, TIR2ΔampR, and CIBT4712. Single strains (*n* = 10) isolated from each of the abovementioned 3 liquid cultivated strains were sequenced at the indicated induction time.

Our findings indicated that we could only adjust the expression of *dacA* at the translational level to balance the production titers and toxicity. Therefore, we randomized the translation initiation region (TIR) of *dacA* as a library for screening optimized strains, including the randomization of the 6 bases before ATG and the 2 amino acid synonymous codons after ATG to form NNNNNNATGN*N*N*N*N*N*. We fused the ampicillin resistance gene to *dacA*, coupling the high-intensity or low-intensity Shine–Dalgarno sequence before the ampicillin resistance gene (*bla*) as a reporter system, and generated 2 kinds of hosts, i.e., strong coupling *bla* (S) and weak coupling *bla* (W). The stronger the translation strength of *dacA*, the greater the ampicillin resistance (Fig. 2A) [22,23].

Then, 5 randomly picked TIR plasmid libraries were sequenced for the RBS sequence, and the results showed that it was 100% randomized (Fig. S1). The plasmid library transformants were extracted with plasmids, and they were transformed into pCas-containing host S or W. The transformants were spread on the corresponding ampicillin-resistant plates. The difference in the number of induced and noninduced strains that formed on the ampicillin-resistant plates in the strain library constructed with the W host was negligible, while the strain library constructed with the S host showed various levels of resistance to ampicillin after induction (Table S9).

Next, 20 transformants were selected from the recombinant S and W strains on the induction and noninduction plates with 50, 100, or 200 μg/ml of ampicillin, and the TIR and the *dacA* coding region were amplified via polymerase chain reaction (PCR; primer: tetR-seq-F/dacA (LcLp)-seq-F) for sequencing. We expected 20 bacterial colonies to show changes only in the TIR, but 4 colonies (TIR2, TIR3, TIR4, and TIR6) had no mutations in *dacA* (Table S10), which indicated that the increase in translation intensity increased toxicity. The strains with no mutations or missense mutations in the *dacA* coding region were selected for fermentation (Table S11). The colonies TIR2, TIR3.1, and TIR25, which showed a greater increase in CDA production, were modified, and their AmpR encoding gene *bla* was knocked out. In TIR2, TIR3.1, and TIR25 without *bla* knockout (KO), the CDA titer was 45 to 60 μM, which was twice the concentration detected in the control CIBT4523, and after knocking out *bla*, the CFUs on the plate were reduced by 2 to 3 orders of magnitude (Fig. 2B and C and Table S12), suggesting that the changes in enzyme structure by fused *bla* deletion might further increase the production of CDA, leading to toxicity and death (Fig. 2B). The sixth amino acid of the AmpR of TIR2, TIR3.1, and TIR25 was further mutated into a stop codon (ampR*). Among them, death of TIR2ampR* (CIBT4712) decreased, CFU increased by an order of magnitude after induction, and the CDA titer was nearly 100% higher than that of CIBT4523 before optimization. However, the *dacA* coding region of TIR3.1ampR* and TIR25ampR* had mutations, and the CDA titer was also very low (Fig. 2C). We compared the mutation rate–time curves of CIBT4523, TIR2ΔampR, and CIBT4712. The 100% mutation occurrence of the 3 strains was 48, 4.5, and 24 h (Fig. 2D), which indicated that the expression toxicity in the 3 strains followed the order CIBT4523 < CIBT4712 < TIR2ΔampR, from weak to strong. Therefore, we selected CIBT4523 and CIBT4712 for further evaluation.

### CIBT4523 and CIBT4712 enhanced the activation of the STING pathway

**Fig. 3.**
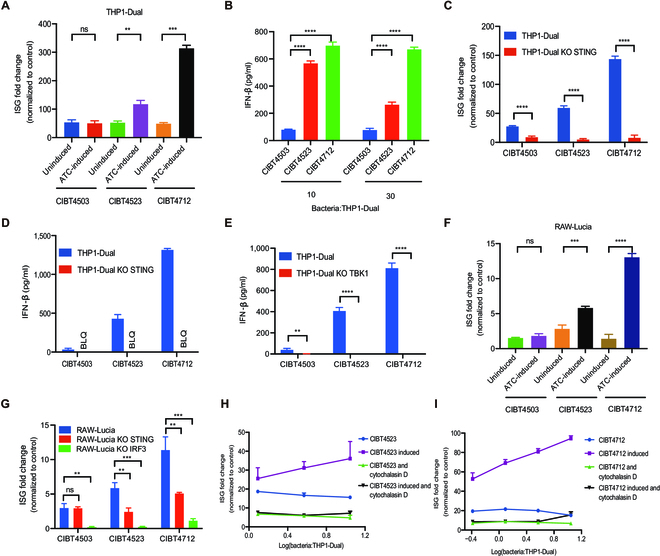
The engineered strains enhanced the activation of the STING pathway in innate immune cells, blocked by cytochalasin D. (A) CIBT4523 and CIBT4712 enhanced the activation of the IFN stimulator gene (ISG) reporter of THP-Dual cells; the ratio of strains to THP1-Dual cells was 10:1. (B) CIBT4523 and CIBT4712 enhanced the production of IFN-β in THP-Dual cells; the ratios of ATC-induced strains to THP1-Dual cells were 10:1 and 30:1. (C) The activation of the ISG reporter induced by the 3 strains was lower in THP1-Dual KO STING cells compared to that in THP1-Dual cells. The ratio of ATC-induced strains to cells was 10:1. (D and E) The IFN-β production induced by the strains was lower in THP1-Dual KO STING and THP1-Dual KO TBK1 cells compared to that in THP1-Dual cells; the ratio of ATC-induced strains to cells was 10:1 (F) CIBT4523 and CIBT4712 increased the activation of the ISG reporter of RAW-Lucia cells; the ratio of strains to RAW-Lucia cells was 30:1. (G) The production of IFN-β induced by the strains was lower in RAW-Lucia KO STING and RAW-Lucia KO IRF3 cells compared to that in RAW-Lucia cells; the ratio of ATC-induced strains to cells was 30:1. (H and I) THP1-Dual cells were pretreated with cytochalasin D (10 μM) or media for 1 h; the ratio of CIBT4523 (H) or CIBT4712 (I) to THP1-Dual cells is shown as indicated. The engineered strains with or without ATC (200 μg/ml) induction were added to the indicated immune cells for 24 h; the fold change of the ISG reporter was calculated relative to that of the control (PBS, phosphate-buffered saline) group, and the secretion of IFN-β was measured. The experiments were conducted in replicates, and the data were expressed as the mean and SD. Statistical significance was compared between different groups by performing Student’s *t* tests; ns, no significant difference. **P < 0.01, ***P < 0.001, and ****P < 0.0001. BLQ, below the limit of quantitation.

To determine the effects of CIBT4523 and CIBT4712 on the activation of the STING pathway, we used THP1-Dual cells, which had the reporters for IFN stimulator genes (ISGs) and NF-κB. We found that the chassis (CIBT4503) could partly activate the ISG pathway in the THP1-Dual cells; however, ATC induction did not enhance the activation of the ISG reporter (Fig. 3A). In contrast, CIBT4523 and CIBT4712 significantly enhanced the activation of the ISG pathway in response to ATC induction (bacteria/THP1-Dual cells = 10); the greatest activation of the ISG reporter was caused by CIBT4712 (Fig. 3A). Thus, compared to CIBT4503, CIBT4523 and CIBT4712 significantly increased the level of IFN-β, a downstream cytokine of the ISG pathway, in THP1-Dual cells under ATC induction (Fig. 3B). We also determined the activation of the NF-κB pathway in THP1-Dual cells and found that all 3 strains effectively activated the NF-κB pathway, with no significant difference in the degree of activation among the different groups (Fig. S2), suggesting that the activation of the NF-κB pathway by the intrinsic antigens of the strain can override the effects of CDA activation.

To confirm whether these engineered bacteria enhanced the ISG pathway by acting on the STING pathway, we used the THP1-Dual KO STING reporter cells, which lacked STING. In the THP1-Dual KO STING cells, activation of the ISG reporter was inhibited in both CIBT4523 and CIBT4712 (Fig. 3C), which indicated that activation of the ISG pathway was STING dependent. Additionally, CIBT4503 lost its ability to activate the ISG reporter in THP1-Dual KO STING cells, suggesting that the chassis can also activate the STING pathway, which might be related to the activation of the upstream cyclic GMP-AMP synthase, as reported in another study [15]. The production of IFN-β induced by all 3 strains was also abolished in the THP1-Dual KO STING cells and THP1-Dual KO TBK1 cells (Fig. 3D and E). These findings further supported the activation of the STING pathway by these strains.

Next, we determined the activation of the STING pathway in RAW-Lucia cells, which are murine macrophages containing an ISG reporter. We found that CIBT4523 and CIBT4712 significantly enhanced the activation of the ISG pathway in RAW-Lucia cells after ATC induction, and CIBT4712 had a stronger effect than CIBT4523 (Fig. 3F). We also examined the activation of the ISG reporter by these strains in RAW-Lucia KO STING cells after ATC induction and found that CIBT4523 and CIBT4712 lost their ability to activate the ISG pathway without STING (Fig. 3G). Next, we detected the activation of the ISG reporter by the 3 strains in RAW-Lucia KO IRF3 cells and found that CIBT4523 and CIBT4712 lost their ability to activate the ISG pathway without IRF3 (Fig. 3G). These results indicated that CIBT4523 and CIBT4712 could activate the STING pathway in murine cells.

### Phagocytosis is required for STING activation

As CDA was mainly present in engineered bacteria, we determined how it activated mononuclear macrophages. Mononuclear macrophages can perform phagocytosis and engulf and lyse bacteria to release CDA, which then activates the STING pathway. To test this, we preincubated THP1-Dual cells with cytochalasin D, a phagocytosis inhibitor, and observed whether the activation of the ISG pathway was blocked by inhibiting phagocytosis. The THP1-Dual cells pretreated with cytochalasin D showed significantly lower activation of the ISG pathway under induced and noninduced conditions of CIBT4523 and CIBT4712 compared to their controls (Fig. 3H and I). These results indicated that CIBT4523 and CIBT4712 activated the STING pathway through phagocytosis. However, the engineered bacteria selectively activated the phagocytic antigen-presenting cells (APCs), unlike small-molecular STING agonists that activate various types of cells.

### CIBT4523 exerted durable antitumor activity and induced immune memory

**Fig. 4.**
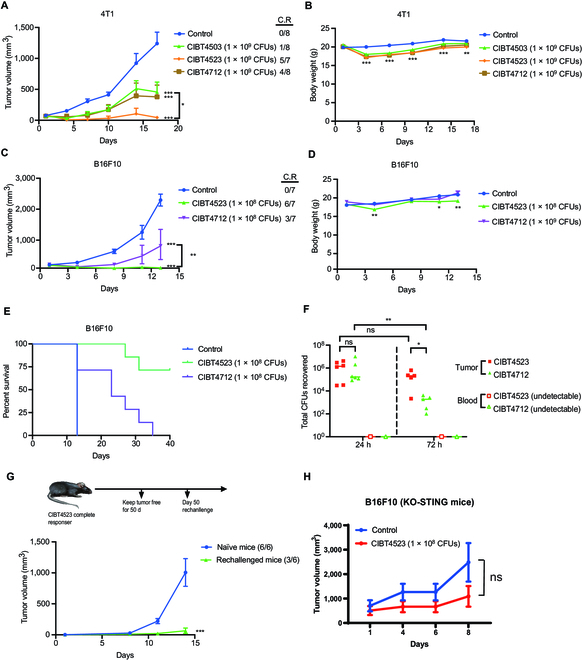
Antitumor activity of the engineered strains in vivo. (A and B) The 4T1 tumor-bearing mice were intratumorally treated with PBS (control), CIBT4503, CIBT4523, or CIBT4712 on days 1, 4, and 7; the tumor growth (A) and body weight (B) were recorded. The number of complete responder (C.R) mice and total mice is shown. (C to E) The B16F10 tumor-bearing mice were intratumorally treated with PBS (control), CIBT4523, or CIBT4712 on days 1, 4, and 7; tumor growth (C), body weight (D), and survival curve (E) are shown. The number of complete responder (C.R) mice and total mice is shown. (F) Total CFUs recovered from B16F10 tumor-bearing mice after injection with CIBT4523 or CIBT4712 (1 × 10^8^ CFUs) for 24 and 72 h are shown; each dot represents a single mouse. (G) Mice were treated with CIBT4523 and kept tumor-free for 50 d. Tumor-free mice or naïve mice were subcutaneously injected with B16F10 cells (1 × 10^5^) in the contralateral flank. The number of B16F10 tumor-bearing mice and total mice are shown. (H) The B16F10 tumor-bearing STING-KO mice were intratumorally treated with PBS (control) or CIBT4523 on days 1, 4, and 7, and tumor growth are shown. *n* = 6. Data were expressed as the mean and SEM. Two-way analysis of variance (ANOVA) was performed to determine differences among groups; ns, no significant difference. *P < 0.05, **P < 0.01, ***P < 0.001, and ****P< 0.0001.

Because CIBT4523 and CIBT4712 can effectively activate STING in vitro, we determined their antitumor activity in immunocompetent mice. The triple-negative mouse breast cancer cell line 4T1 is resistant to programmed cell death-1/ligand 1 antibody therapy. Thus, we used this cell line as an allogenic in situ tumor model. The 4T1 tumors were intratumorally injected with 3 strains on days 1, 4, and 7. The results showed that CIBT4503, CIBT4523, and CIBT4712 significantly inhibited the growth of 4T1 tumors (Fig. 4A). Specifically, CIBT4523 showed the strongest antitumor activity with a TGI (tumor growth inhibition) of 103%, which achieved complete tumor regression in 5 of 7 mice, followed by CIBT4712 (73% TGI), which achieved complete tumor regression in 4 of 8 mice. The control strain CIBT4503 (66% TGI) showed the weakest antitumor activity, with complete tumor regression in only 1 of 8 mice. The weight of the animals decreased after treatment with either of the 3 strains, and it gradually increased with time in the animals of all 3 treatment groups (Fig. 4B). These results showed that the engineered strains CIBT4523 and CIBT4712 had significantly higher antitumor activity. Unlike the activation of the STING pathway in vitro, CIBT4523 showed stronger antitumor activity than CIBT4712 in the tumor model. Similarly, CIBT4523 had stronger antitumor activity than CIBT4712 in the mouse B cell lymphoma A20 tumor model. Although the difference in the inhibition of tumor growth was not significant, the tumor regression rate was higher in the CIBT4523 group relative to the CIBT4712 group or CIBT4503 group (Fig. S3A). The weight of the animals decreased after treatment with all 3 strains, and it gradually increased with time in the animals of all 3 treatment groups (Fig. S3B).

Next, we compared the antitumor effect of CIBT4523 and CIBT4712 in the subcutaneous B16F10 melanoma allograft model in C57 mice. The B16F10 allograft tumors were intratumorally injected with CIBT4523 and CIBT4712 on days 1, 4, and 7. The results showed that CIBT4523 had a stronger antitumor effect and showed a higher rate of tumor regression (6 of 7) than CIBT4712 (Fig. 4C), and it slightly decreased the weight of the mice (Fig. 4D). We also evaluated the effect on the survival time of mice. Compared to the mice in the control group, those in the CIBT4523 treatment group had a significantly longer survival time, followed by the survival time of the mice in the CIBT4712 treatment group (Fig. 4E).

We also investigated why CIBT4712 had lower antitumor activity than CIBT4523 in vivo although it showed a stronger effect on immune activation than CIBT4523 in vitro. As CIBT4712 can cause bacterial lysis because of excessive CDA production, as shown above, we analyzed whether the same problem occurred in tumor tissues. After intratumoral injection of bacteria for 24 and 72 h, we collected the whole tumor tissue and counted the number of viable bacteria. The number of viable CIBT4523 and CIBT4712 bacteria was similar after 24 h (Fig. 4F). However, the number of CIBT4712 was significantly lower than that of CIBT4523 after 72 h of treatment (Fig. 4F), which suggested that the survival rate of CIBT4712 in the tumor tissue was lower than that of CIBT4523. This might have decreased the antitumor efficacy. We also analyzed the levels of CIBT4523 and CIBT4712 in the blood and did not detect any bacteria (Fig. 4F), which indicated that these bacteria were mainly localized in the tumor and did not cause systemic toxicity.

We determined whether CIBT4523 treatment could induce immune memory to reject rechallenged tumors. Six mice of the B16F10 tumor model that were cured by CIB4523 treatment were fed for another 50 d without tumor recurrence. Then, we reinoculated the B16F10 tumor cells into these mice, while naive mice that were not previously inoculated with tumors were used as control. We found that all 6 control mice developed tumors after inoculation with B16F10 cells, while only half of the mice in the rechallenged group developed tumors. The tumor growth rate of the mice in the treatment group was considerably lower than that of the mice in the control group (Fig. 4G and Fig. S3C). These results suggested that immune memory could be induced in the mice cured by CIBT4523 to reject tumor rechallenge. Three mice of the A20 tumor model that were cured by CIBT4523 treatment were reinoculated with A20 after 2 months of tumor-free survival. All treated mice were resistant to tumor reinoculation, whereas tumor growth was observed in all control mice (Fig. S3D), which indicated that CIBT4523 treatment also induced immune memory in A20 tumors. Additionally, we assessed whether the antitumor effect of CIBT4523 was through the STING protein in vivo. For this, the B16F10 melanoma model was established in STING-KO C57BL/6 mice. We found that the antitumor effect of CIBT4523 was not significant in the STING-KO mice compared to the control (Fig. 4H), which was considerably lower than that in the wild-type C57BL/6 mice (Fig. 4C). This result suggested that the antitumor effect of CIBT4523 was mainly through the STING protein in vivo.

### CIBT4523 reinforced antitumor immunity in vivo

**Fig. 5.**
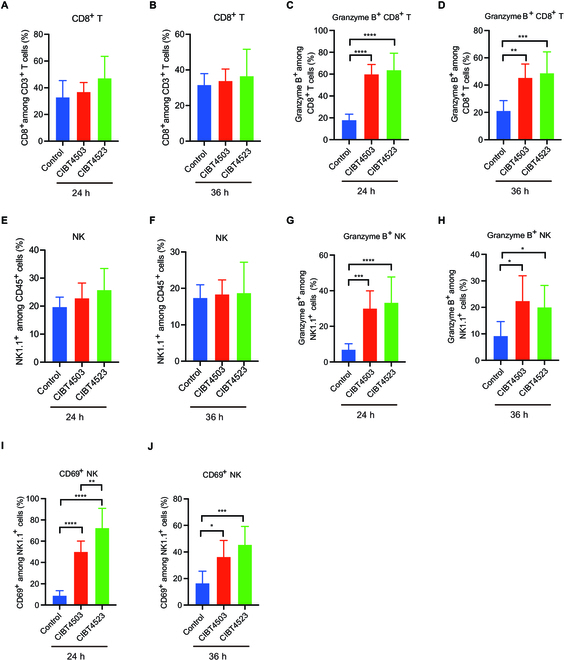
CIBT4523 enhanced the antitumor immune microenvironment. (A to J) The proportion of CD8^+^ T cells (A and B), granzyme B^+^ CD8^+^ T cells (C and D), natural killer (NK) cells (E and F), granzyme B^+^ NK cells (G and H), and CD69^+^ NK cells (I and J) in B16F10 tumor tissues of control, CIBT4503-treated, and CIBT4523-treated groups. The B16F10 tumor-bearing mice were intratumorally treated with PBS, CIBT4503, or CIBT4523 (1 × 10^8^ CFUs) for 24 or 36 h. Then, the tumor tissues were isolated, and the infiltrating immune subsets were analyzed by flow cytometry (*n* = 6). One-way ANOVA was performed to determine differences among groups; *P < 0.05, **P < 0.01, ***P < 0.001, and ****P < 0.0001.

Because CIBT4523 exhibited higher antitumor efficacy and CDA production (Fig. S4) than its chassis control CIBT4503 in vivo, we next determined whether it has a higher antitumor immunity. Flow cytometry assays were performed to detect changes in cytotoxic immune cell subtypes, including CD8^+^ T cell and natural killer (NK) cell subsets, in tumor tissues after 24 or 36 h of treatment with CIBT4523 and CIBT4503. The frequency of CD8^+^ T cells did not change significantly in CIBT4523-treated and CIBT4503-treated groups after 24 or 36 h of treatment (Fig. 5A and B), while the cytotoxic immune subset granzyme B^+^ CD8^+^ T cells increased significantly after treatment with CIBT4523 and CIBT4503 (Fig. 5C and D). The number of cells was slightly higher after 36 h of treatment with CIBT4523 (Fig. 5D). Similarly, NK cells did not change significantly in the CIBT4523-treated and CIBT4503-treated groups (Fig. 5E and F), while the cytotoxic immune subsets, including granzyme B^+^ NK and CD69^+^ NK, increased significantly after treatment with CIBT4523 and CIBT4503. The increase was slightly higher after treatment with CIBT4523 than after treatment with CIBT4503 (Fig. 5G to J). These findings indicated that CIBT4523 increased antitumor immunity more effectively than CIBT4503 in vivo.

**Fig. 6.**
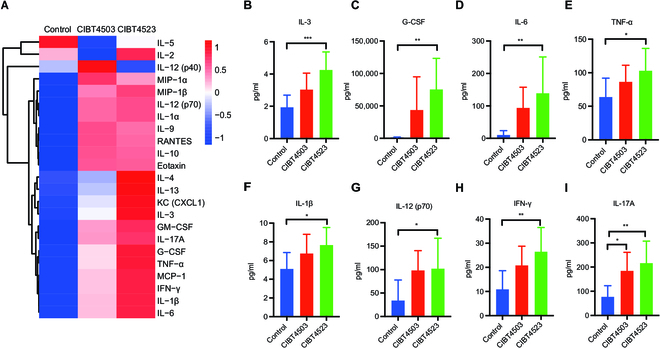
CIBT4523 increased the levels of immune-activating cytokines in the plasma. B16F10 tumor-bearing mice were intratumorally treated with PBS, CIBT4503, or CIBT4523 (1 × 10^8^ CFUs) for 6 h, and the concentrations of plasma cytokines were measured (*n* = 8). (A) A heatmap of the expression of 23 cytokines in the plasma is shown as the mean expression of cytokines in each group. (B to I) The concentration of cytokines in 3 groups; the significant differences among groups were determined by one-way ANOVA with Tukey’s multiple comparisons tests; *P < 0.05, **P < 0.01, and ***P < 0.001. The mean and SD are shown.

Next, we determined the production of plasma cytokines in CIBT4523-treated and CIBT4503-treated mice, which is functionally associated with the activation of antitumor immunity. A cytokine array was used to evaluate the plasma levels of 23 cytokines after treatment with CIBT4523 and CIBT4503. The results showed that the levels of many cytokines increased after treatment with CIBT4523 and CIBT4503; specifically, CIBT4523 treatment exhibited considerably higher induction of many cytokines (Fig. 6A). We also found that interleukin-3 (IL-3) and granulocyte colony-stimulating factor (G-CSF), which promote the proliferation of myeloid cells, increased significantly after CIBT4523 treatment, while treatment with CIBT4503 only showed an increasing trend (Fig. 6B and C). Additionally, GM-CSF (granulocyte-macrophage CSF) also increased significantly after treatment with CIBT4523 and CIBT4503, with a slightly higher increment after treatment with CIBT4523 (Fig. S5A). The levels of IL-6, tumor necrosis factor–α (TNF-α), and IL-1β, which have proinflammatory and immune activation functions, also increased significantly after CIBT4523 treatment but not after CIBT4503 treatment (Fig. 6D to F). In contrast, the levels of macrophage inflammatory protein 1α (MIP-1α), MIP-1β, RANTES, and eotaxin, which have chemotaxis functions, increased significantly after CIBT4523 and CIBT4503 treatment, and keratinocyte chemoattractant/(CXCL1) increased only after CIBT4523 treatment (Fig. S5B to F). The levels of IL-12 (p70) and IFN-γ, which are associated with T cell function, also increased significantly after CIBT4523 treatment but did not increase significantly after CIBT4503 treatment (Fig. 6G and H). The levels of IL-17A also increased significantly after treatment with CIBT4523 and CIBT4503, and CIBT4523 induced a slightly higher increment (Fig. 5I). To summarize, the above results indicated that CIBT4523 has higher proinflammatory and immune activation functions than CIBT4503, which is consistent with the changes in the tumor immune microenvironment. Therefore, the stronger immune activation function of CIBT4523 compared to that of the chassis CIBT4503 might account for its better antitumor activity in vivo.

## Discussion

In this study, we first determined the effect of the level of CDA expression on the control of autotoxicity and antitumor efficacy. We designed biotherapeutic strains to produce the STING agonist CDA based on the nutrition-deficient strain CIBT4503. After multiple optimization strategies, we obtained 2 stable strains, CIBT4523 and CIBT4712, with high CDA production by optimizing the *dacA* codon. The CIBT4523 and CIBT4712 strains enhanced the activation of the STING pathway in human and mouse phagocytic cells after ATC induction more effectively than the CIBT4503 strain. The CDA produced by bacteria entered the target cells via phagocytosis, which indicated the selectivity of these biotherapeutic agents in phagocytes. We also found that the antitumor activity of CIBT4523 and CIBT4712 was higher than the chassis CIBT4503 in allograft tumor models of immunocompetent mice, including breast cancer (4T1), B cell lymphoma (A20), and melanoma (B16F10). Specifically, CIBT4523 showed the strongest antitumor potency and induced immune memory against rechallenged tumors. We also found that the enhanced antitumor activity of CIBT4523 was associated with a more potent activation of antitumor immunity.

We selected the EcN strain because it preferentially aggregated in tumor tissue more effectively than other strains in the intravenous administration experiments that were conducted with animal models [24,25]. Additionally, EcN has been safely used for nearly 100 years as a pharmaceutical ingredient in multiple licensed medicinal products with anti-inflammatory and antimicrobial activities [26–28]. We incorporated 2 auxotrophies, i.e., *thyA* and *dapA*, into the strains that we designed for safety and biocontainment. This made the strains incapable of replicating in the tumor microenvironment and incapable of surviving outside the tumor microenvironment. Additionally, for safety, all antibiotic-resistant genes were removed or translation was terminated from the engineered strains so that they might be eliminated by available treatments.

CDA is an essential signaling molecule in many bacteria and archaea [29] and is usually required under normal growth conditions [30]. However, excessive accumulation of CDA in host bacterial cells can lead to toxicity [21,31]. Therefore, a balance is required between host cell growth and CDA production, which is also crucial for EcN. We used the non-*E. coli* codon of the *dacA* gene to weaken translation and obtain the strain CIBT4523 with high CDA yield. We also obtained the strain CIBT4712, which had twice the CDA yield but controllable toxicity, by fusing *dacA* and the ampicillin-resistant reporter gene. These 2 strains showed a balance between CDA production and toxicity in vitro.

According to the toxicity of CDA, tightly regulated inducible expression platforms are necessary for *dacA* expression. We selected tetracycline-regulated systems, which utilize the constitutively expressed repressor TetR that binds to the tetO operator sequence in the promoter and prevents RNA polymerase from binding to DNA and initiating transcription [32]. We selected this system because the synthetic inducer ATC is not found in bacterial growth media, the mouse intestine, food items, or the human and mouse microbial communities, which allowed us to precisely control the expression of the gene in vivo, allowing on-demand delivery of therapeutic compounds. The use of the synthetic inducer ATC to alter gene expression does not perturb the dynamics of microbial metabolism, growth, and community composition, unlike the case after the administration of dietary polysaccharides or sugars [33]. However, further research on the orthogonalization of natural inducers, such as arabinose, which disrupt the effect of glucose, also needs to be conducted [34].

The CIBT4503 strain partly activated the STING pathway, which might be related to the activation of the upstream cGAS by bacterial DNA [15]. In contrast, our engineered strains CIBT4523 and CIBT4712 exhibited considerably higher induction of the STING pathway under ATC induction, as shown by the increased production of the ISG reporter and IFN-β. The CIBT4712 strain showed higher activation of the ISG pathway than the CIBT4523 strain at the cellular level, which was consistent with the production of CDA in vitro. We also found that the CDA of CIBT4523 and CIBT4712 entered phagocytes mainly through phagocytosis and then activated the STING pathway. This suggested that these strains selectively activated phagocytes and did not activate any other type of cell, unlike small-molecule STING agonists that induce systemic activation. This is a unique advantage of using living agents.

Although CIBT4712 showed higher CDA induction and activation of the STING pathway than CIBT4523, CIBT4712 showed lower antitumor efficacy in allograft tumor models than CIBT4523. To determine the reason for the lower antitumor activity of CIBT4712, we evaluated the number of viable bacteria in tumor tissues and found that the number of bacteria in the tumor of CIBT4712 decreased substantially at 72 h compared to that at 24 h, while CIBT4523 maintained the number of bacteria. This finding suggested that the high CDA yield in CIBT4712 might result in self-toxicity and decreased bacterial vitality, which, in turn, can reduce immune activation in the tumor microenvironment. Therefore, the optimum expression of CDA in engineered strains is essential for balancing antitumor efficacy and self-toxicity. We found that the antitumor effect of CIBT4523 was favorable and that the cured mice were resistant to rechallenged tumors, which suggested that CIBT4523 can trigger innate and adaptive immunity to induce tumor regression [35–37]. Furthermore, we found that the activation of ISG signaling induced by the engineered strains was abolished in STING-KO or TBK1-KO THP1-Dual cells, as well as STING-KO or IRF3-KO RAW-Lucia cells. Additionally, the antitumor effect observed in vivo was obstructed in STING-KO mice. These results indicated that the CDA generated by the engineered strain plays a critical role in STING's antitumor function

To elucidate the immunological mechanisms of CIBT4523 in vivo, we initially analyzed the activation of the tumor immune microenvironment and the secretion of cytokines in the blood. We focused on the changes in the 2 classical cytotoxic immune subsets, i.e., CD8^+^ T and NK cell subsets, although the proportion of CD8^+^ T cells and NK cells did not change significantly after treatment with either CIBT4503 or CIBT4523, while the functionally activated subsets of CD8T cells (granzyme B^+^ CD8^+^ T) and NK cells (granzyme^+^ NK and CD69^+^ NK) [38,39] increased substantially after treatment with CIBT4503 and CIBT4523, with higher induction after CIBT4523 treatment. These results indicated that both CIBT4523 and CIBT4503 had an immune-activating effect on the tumor microenvironment, and CIBT4523 exhibited considerably higher antitumor immunity than CIBT4503. Additionally, CIBT4523 treatment induced a higher production of immune-activating plasma cytokines, especially IL-3, G-CSF, TNF-α, IL-1β, IL-12, and IFN-γ, which were not increased by CIBT4503 treatment. This finding further confirmed the stronger immune-activating effect of CIBT4523. For example, IL-3 and G-CSF are GM-CSFs that promote the production, differentiation, and function of various immune cells [40,41], and TNF-α, IL-1β, IL-12, and IFN-γ are potent cytokines that activate the immune system and elicit antitumor responses [42–45]. Their concurrent increase might synergistically increase antitumor immunity.

To summarize, the optimum expression of CDA in engineered strains is essential for balancing antitumor efficacy and self-toxicity. We successfully engineered a strain (CIBT4523) with optimal CDA production, which effectively reinforced the antitumor immune response and potency in vivo. These results indicated that CIBT4523 has a high translational potential in antitumor immunity, and combining CIBT4523 with other immunotherapies, such as anti-PD-1/L1 therapy, might be a promising treatment strategy [46].

## Materials and Methods

### Strains, plasmids, and growth conditions

All plasmids were introduced by transformation into *E. coli* DH5α or Trans1-T1 phage-resistant chemically competent cells (TransGen, Beijing) for cloning or into EcN for expression or genome editing and maintained in Luria–Bertani broth (LB); they were incubated at 37 °C with appropriate antibiotics when necessary (25 mg/l of kanamycin and 50 mg/l of spectinomycin). Agar (15 g/l) was added for plating. All recombinant strains were transformed by electroporation. All strains were stored as glycerol stocks at −80 °C in 20% glycerol LB. The strains and plasmids used in the study are shown in Table S13. The corresponding genes were synthesized using GenScript (Nanjing, China).

### Plasmid cloning, strain genome editing, and construction of the TIR library strain

Plasmids, genomic DNA, and DNA were extracted or purified using AxyPrep kits (Corning, USA). The Taq (Thermo Scientific, USA) or KOD-plus-neo polymerases (Toyobo, Japan) were used for performing PCR. The pTargetF series (Table S13), used in targeting single-gene modifications with a targeting N20 sequence for the gene loci of interest, was obtained by performing inverse PCR with the modified N20 sequence hanging at the 5′ end of the primers (listed in Table S14), followed by self-ligation. The plasmids were cloned using restriction endonucleases, T4 DNA ligase (Thermo Scientific, USA), and the isothermal assembly method (details in Table S15) [47]. The corresponding primers used are listed in Table S14. The RBSs expressing *dacA* controlled by Ptet were optimized using the web interface RBS online calculator (https://salislab.net/) to achieve the targeted translation initiation rate. The optimized sequences of RBSs are listed in Table S7. The genome editing of EcN was performed using CRISPR/Cas [48] using plasmids with or without donor DNAs, listed in Table S16.

The pTargetT-TIR library was constructed by assembling the fragments consisting of degenerate TIR sequences around the start codon of *dacA* (5′NNNNNNATGGAYTTY3′) generated by annealing 2 degenerate primers ptet-TIR-dacA (LcLp)-F1 and dacA (LcLp)-TIR-ptet-R, the upstream and downstream homologous fragments to the host W/S, and the plasmid backbone generated from EcoRI/HindIII-digested pTargetF-RBSdacA (details in Table S16). The pTargetT-TIR library was transformed into Trans1-T1 to test the quality of the library by sequencing the TIR region of the transformants. The transformants on the pTargetT-TIR library plate (about 10^5^ transformants in 10 plates) were washed and scraped off with 4 ml of LB medium, and the plasmids were extracted in 10 tubes, 30 μl each. Then, 10 μl of the pTargetT-TIR plasmid was transformed into the host S and W competent cells containing pCas and cultured in LB medium containing 50 mg/l of spectinomycin, 25 mg/l of kanamycin, 100 μg/ml of 2,6-diaminopimelic acid, and 3 mM thymine. All colonies were scraped off and transformed in LB medium containing 100 μg/mL of 2,6-diaminopimelic acid, 3 mM thymine, and ampicillin (12.5, 25, 50, 100, 200, 400, 800, 1,600, and 3,200 μg/ml) with or without induction using ATC (200 ng/ml).

### Strain fermentation and CFU detection

The strains stored in the −80 °C stock were inoculated in LB tubes containing 100 μg/ml of 2,6-diaminopimelic acid and 3 mM thymine (with appropriate antibiotics). The cells were cultured for about 16 h in a shaker at 37 °C and 250 rpm. Then, 1% (v/v) of the liquid strain was inoculated in 250-ml flasks with 20 ml of 2YT medium containing 2,6-diaminopimelic acid and thymine and incubated in a shaker for 1.5 to 2.0 h at 37 °C and 250 rpm until the optical density at 600 nm (OD_600_) was about 0.8 to 1.0. Next, 200 ng/ml of dehydrotetracycline was added for induction for 4.5 h. The CFUs were then calculated by diluting the liquid strain (10^3^, 10^5^, and 10^7^) with phosphate-buffered saline (PBS) and spreading it on plates for overnight incubation.

### CDN detection in vitro

First, 1 ml of the fermented sample was centrifuged at 13,000 rpm for 10 min. Then, the supernatant was removed, and the remaining sample was stored at −20 °C for further analysis. The strain samples were resuspended and lysed by lysozyme. The cell lysate was extracted, dried, and resuspended in sterile water and analyzed by liquid chromatography-mass spectrometry [30].

### ISG and NF-κB reporter assay

THP1-Dual cells (thpd-nfis, InvivoGen, French) containing both ISG-luciferase and NF-κB-alkaline phosphatase (SEAP) reporters were used. THP1-Dual KO STING cells (thpd-kostg, InvivoGen) were knockout STING THP1-Dual cells. THP1-Dual KO TBK1 cells (thpd-kotbk, InvivoGen) were knockout TBK1 THP1-Dual cells. RAW-Lucia ISG cells (rawl-isg, InvivoGen) were murine macrophages containing an ISG-luciferase reporter. RAW-Lucia KO STING cells (rawl-kostg, InvivoGen) were STING-KO RAW-Lucia ISG cells. RAW-Lucia KO IRF3 (rawl-koirf3, InvivoGen) were IRF3 knockout RAW-Lucia ISG cells. All cells were maintained following the manufacturer's recommendations and incubated at 37 °C in a humidified atmosphere containing 5% CO_2_ and 95% air.

The cells were plated in 96-well plates (catalog: 3601; Corning) at 1 × 10^5^ cells per well (THP1-Dual cells, THP1-Dual KO STING cells, and THP1-Dual KO TBK1 cells) or 5 × 10^4^ cells per well (RAW-Lucia and RAW-Lucia KO IRF3) in 180 μl of the medium. Each engineered strain with or without ATC induction was collected and counted, as well as resuspended in 2 ml of PBS buffer. Then, media containing bacteria (at the indicated ratios) or alone were added to 96-well cell culture plates to reach a total volume of 200 μl. After 24 h, 20 μl of the cell supernatant and 50 μl of the QUANTI-Luc (rep-qlc1, InvivoGen) detection reagent (ISG reporter assay) or 200 μl of the QUNTI-Blue (rep-qbs, InvivoGen) SEAP detection reagent (NF-κB reporter assay) were added to a 96-well black plate. The luciferase activity and absorbance were measured by SpectraMAX Paradigm or SpectraMAX Plus 384 (Molecular Devices, Sunnyvale, CA). Additionally, to perform the cytochalasin D (GC13440, GlpBio, US) assay, these compounds (10 μM) were pretreated with the indicated cells for 1 h before each strain was added.

### Determining IFN-β levels

First, cells were seeded in 96-well plates at a density of 1 × 10^5^ cells per well and treated with the indicated strains (bacteria/cells = 10) for 24 h. The cell supernatant was collected, and the expression of IFN-β was tested using the test kit (70-EK1236–96, Dakewe, China). The cytokine concentration was calculated on the basis of the standard curve. The test was conducted in triplicate.

### Animal studies

Immunocompetent C57BL/6 and BALB/c female mice (6 to 8 weeks old) were purchased from Beijing Vital River Laboratory Animal Technology Co. Ltd. STING-KO C57BL/6 mice (8 to 10 weeks old) were purchased from GemPharmatech (Nanjing, China). All animal experiments were performed following the institutional ethical guidelines on animal care and were approved by the Institute Animal Care and Use Committee at Shanghai Institute of Materia Medica. The B16F10 melanoma (CRL-6475) cells, A20 B cell lymphoma (TIB-208) cells, and 4T1 mammary carcinoma (CRL-2539) cells were obtained from the American Type Culture Collection.

The 4T1 cells (2.5 × 10^5^) were inoculated into the breast fat pad of BALB/c mice. The A20 cells (5 × 10^5^) were subcutaneously injected into BALB/c mice. The B16F10 (1 × 10^5^) cells were subcutaneously injected into C57BL/6 mice or STING-KO C57BL/6 mice. When the tumors reached the indicated volume, the mice were randomized into different groups. Each mouse was intraperitoneally injected with 60 μl of ATC (200 μg/ml), and 4 h later, 100 μl of the indicated dose of bacteria or vehicle (PBS) was injected into the tumors. The treatment was administered on days 1, 4, and 7. The tumor volume and body weight were measured twice a week. The formula for calculating tumor volume was *V* = length × width^2^/2. For the survival analysis, mice with tumors that exceeded 2,000 mm^3^ were euthanized. Complete responder mice were monitored for at least 50 d with no relapse.

### Quantification of bacteria in tumor tissues

The B16F10 (1 × 10^5^) cells were subcutaneously injected into C57BL/6 mice. When the tumors reached a volume of 300 to 500 mm^3^, the mice were randomized into different groups, as indicated. The mice were intraperitoneally injected with 60 μl of ATC (200 μg/ml), and 4 h later, 100 μl of CIBT4523 (1 × 10^8^ CFUs), CIBT4712 (1 × 10^8^ CFUs), or vehicle (PBS) was administered. After 24 or 72 h, the mice were euthanized, and the tumors were removed via dissection. The harvested tumors were placed in sterile bead bug homogenizer tubes, prefilled with 6-mm stainless steel beads (JX-GZ0139, JINGXIN, China), containing 500 μl of sterile PBS. The tumors were homogenized for 2 min using a tissue grinding machine (Scientz-48, China). Whole tumor homogenates were used for quantifying bacterial CFUs.

### Analysis of immune cell subsets in tumor tissues and cytokines in the plasma

When the volume of the B16F10 tumors reached 300 to 500 mm^3^, C57BL/6 mice were intraperitoneally injected with 60 μl of ATC (200 μg/ml) for 4 h and then intratumorally treated with PBS, CIBT4503 (1 × 10^8^ CFUs), or CIBT4523 (1 × 10^8^ CFUs) for 24 or 36 h. The tumors were cut into pieces and incubated in 2 ml of the 1640 medium containing 0.1% type IV collagenase (LS004188, Worthington, USA) and 0.01% deoxyribonuclease I (10104159001, Roche, Swiss) at 37 °C for 25 min. Single cells were filtered through a 40-μm cell strainer and transferred to a 15-ml tube. Next, the cells were resuspended in 3 ml of red blood cells lysis buffer and incubated at room temperature for 5 min to remove red blood cells. Then, the cells were counted using a hemocytometer. The cells were stained with the LIVE/DEAD cell stain kit (565388, BD, USA) for 10 min in the dark, and then, they were resuspended in 100 μl of magnetic-activated cell sorting buffer and stained with surface antibodies, including CD45-BUV395 (catalog: 564279, BD), CD45-FITC (catalog: 103108, BioLegend, USA), CD3-FITC (catalog: 555274, BD), CD3-percp-cy5.5 (catalog: 561108, BD), CD8-BUV737 (catalog: 612759, BD), NK1.1-APC (catalog: 108710, BioLegend), NK1.1-percp-cy5.5 (catalog: 108728, BioLegend), CD69-BUV395 (catalog: 569367, BD), and CD16/CD32 (catalog: 553141, BD). For intracellular protein staining, the eBioscience Foxp3|Transcription Factor Staining Buffer kit (00–5523–00, Thermo, USA) was used to fix and permeabilize cells and then resuspended with antibodies, including granzyme B-BV421 (catalog: 396414, BioLegend). The cells were then fixed with 500 μl of 4% paraformaldehyde in the dark for 15 min. Finally, the cells were resuspended in 350 μl of magnetic-activated cell sorting buffer and kept at 4 °C until they were analyzed by flow cytometry (BD LSRFortessa). All data were analyzed using the FlowJo software. For detecting cytokines, a cell stimulation cocktail (catalog: 00–4975–03, Thermo) was added 4 h before staining.

### Determination of plasma cytokines

When the volume of B16F10 tumors reached 300 to 500 mm^3^, C57BL/6 mice were intraperitoneally injected with 60 μl of ATC (200 μg/ml) for 4 h and then intratumorally treated with PBS, CIBT4503 (1 × 10^8^ CFUs), or CIBT4523 (1 × 10^8^ CFUs) for 6 h. The mice were anesthetized, plasma was collected, and cytokines were determined using the Bio-Plex Pro Mouse Cytokine Grp I Panell (#M60009RDPD, Bio-Rad, USA) kit following the manufacturer's protocol.

### Data analysis

The Prism7.0c software (GraphPad Software, San Diego, CA) was used for conducting all statistical analyses.

## Data Availability

Data are available on reasonable request. The datasets generated during the current study are available from the corresponding authors on reasonable request.
