# Supplementary material for: Fine-tuning Bacterial Cyclic di-AMP Production for Durable Antitumor Effects Through the Activation of the STING Pathway
Source: Research (Wash D C). 2023 Mar 30;6:0102. doi: 10.34133/research.0102 (PMC10065788; doi:10.34133/research.0102)
Supplement: Supplementary 1 — Tables S1 to S16 Figs. S1 to S5 [file research.0102.f1.docx]

**Supplemental Tables**

**Contents**

Table S1. CDNs production by recombinant EcN under control of Ptet.

Table S2. *dacA* mutation in recombinant EcN

Table S3. CFUs of recombinant EcN expressing *dacA* under different conditions

Table S4. The effect of ATC induction time on CDA production in CIBT4523

Table S5. The effect of ATC concentration on CDA production in CIBT4523

Table S6. CFUs of recombinant EcN expressing *dacA* with different gene dosages

Table S8. Genotype and phenotypes of RBS optimized strains

Table S9. CFUs of RBS library on plates with different ampicillin concentrations

Table S10. TIR and *dacA* mutation in RBS library selected by ampicillin

Table S11. CDA production from selected strains

Table S12. Strains for CDA production

Table S13. Strains and plasmids

Table S14 Primers used in the study

Table S15. Materials and methods used for plasmids cloning in this study

Table S7. RBS sequence variation expressing *dacA* based on Salis online software

Table S16. Materials used in recombinant strains constructions by CRISPR/Cas method

**Table S1. CDNs production by recombinant EcN under control of Ptet**

| **CDN** | **Recombinant EcN** | **Titer w/o induction (μM)** | **Titer with induction (μM)** |
| --- | --- | --- | --- |
| cyclic di-AMP | CIBT8783 | 10.4 | 0.2 |
| 3’3’-cyclic GMP-AMP | CIBT8784 | 0 | 1.75 |
| cyclic di-GMP | CIBT8785 | 0 | 3.5 |

**Table S2. *dacA* mutation in recombinant EcN**

| Strains | *dacA* mutation |
| --- | --- |
| lacZ:dacAec1 | 665G>T |
| lacZ:dacAec2 | 452_770del |
| lacZ:dacAec3 | 33_221del |
| lacZ:dacAec4 | 452_770del |
| lacZ:dacAec5 | 521_522ins |
| lacZ:dacAec6 | 263C>A |

**Table S3. CFUs of recombinant EcN expressing *dacA* under different conditions**

| With or w/o induction | ATC concentration (ng/ml) | 0 h | | 1 h | | 3 h | | 5 h | |
| --- | --- | --- | --- | --- | --- | --- | --- | --- | --- |
|  |  | OD_600_ | CFU/ml | OD_600_ | CFU/ml | OD_600_ | CFU/ml | OD_600_ | CFU/ml |
| w/o | 0 | 0.296 | 9×10^7^ | 1.535 | 1×10^9^ | 2.475 | 4.5×10^9^ | 2.510 | 4×10^9^ |
| w | 10 | 0.377 | 2.9×10^8^ | 0.905 | 8×10^5^ | 0.995 | 1.1×10^7^ | 1.190 | 6.5×10^7^ |
|  | 20 | ND | ND | 0.740 | 4×10^5^ | 0.930 | 1.3×10^7^ | 1.185 | 1.2×10^8^ |
|  | 40 | ND | ND | 0.895 | 1×10^6^ | 1.040 | 1.1×10^7^ | 1.210 | 6.3×10^7^ |
|  | 80 | ND | ND | 0.615 | 6 | 0.760 | 9.9×10^6^ | 1.080 | 2.3×10^8^ |
|  | 100 | ND | ND | 0.565 | 6×10^5^ | 0.710 | 1.6×10^7^ | 1.105 | 2.5×10^8^ |
|  | 150 | ND | ND | 0.575 | 8×10^5^ | 0.805 | 1.4×10^7^ | 1.110 | 5×10^7^ |
|  | 200 | ND | ND | 1.095 | 1.7×10^6^ | 1.265 | 1.5×10^7^ | 1.355 | 7×10^7^ |

**Table S4.** **The effect of ATC induction time on CDA production in CIBT4523**

| ATC concentration (ng/ml) | 2h | | | 4h | | | 6h | | | 10h | | |
| --- | --- | --- | --- | --- | --- | --- | --- | --- | --- | --- | --- | --- |
|  | OD600 | CFU | CDA (μM) | OD600/ml | CFU/ml | CDA (μM) | OD600 | CFU/ml | CDA (μM) | OD600 | CFU/ml | CDA (μM) |
| 0 | 4.35 | 1.9×10^8^ | 0.00 | 4.97 | 6.2×10^8^ | 0.84 | 5.11 | 9.5×10^8^ | 0.72 | 4.35 | 5.0×10^7^ | 0.37 |
| 200 | 3.99 | 2.76×10^9^ | 1.44 | 5.20 | 1.18×10^9^ | 11.43 | 5.17 | 7.6×10^8^ | 8.35 | 4.19 | 4.0×10^7^ | 4.99 |

**Table S5.** **The effect of ATC concentration on CDA production in CIBT4523**

| ATC concentration (ng/ml) | OD600 | CFU/ml | CDA (μM) |
| --- | --- | --- | --- |
| 0 | 5.38 | 5.2×10^8^ | 0.79 |
| 100 | 5.25 | 1.26×10^9^ | 11.93 |
| 200 | 5.21 | 7.5×10^8^ | 11.57 |
| 300 | 5.05 | 3.39×10^9^ | 9.65 |
| 400 | 5.21 | 1.18×10^9^ | 11.19 |

**Table S6. CFUs of recombinant EcN expressing dacA with different gene dosages**

|  | With or w/o ATC induction | CFU/ml |
| --- | --- | --- |
| CIBT4523 | w/o | 4.15±3.85×10^9^ |
|  | w | 4.10±3.90×10^9^ |
| CIBT8786-plasmid expressing with pMB1 replicon | w/o | 5.00±2.24×10^9^ |
|  | w | 4.13±4.06×10^5^ |
| CIBT8787-plasmid expressing with pSC101 replicon | w/o | 6.00±1.00×10^9^ |
|  | w | 1.83±0.81×10^9^ |
| CIBT4524-integrating double copies | w/o | 6.50±0.50×10^9^ |
|  | w | 4.50±2.29×10^9^ |
| CIBT8788-integrating triple copies | w/o | 1.83±0.24×10^8^ |
|  | w | 1.30±0.50×10^8^ |

**Table S7. RBS sequence variation expressing *dacA* based on Salis online software***

| Predicted translation rates (strains) | RBS sequence (5’ to 3’) |
| --- | --- |
| R385 (CIBT4523) | AGGAGGTCCACTATTTG**ATG** |
| R300 | CCCATCGGAGTTTTAGGCAGCTTAAAC**ATG** |
| R725 | CCCATCGGAGTTTTATGGAGCTTTAAC**ATG** |
| R1099 (CIBT4707) | CCCATCGGAGTTTTAAGGAGCTTTGAT**ATG** |
| R1637 | CCCATCGGAGTTTTAGGCAGGTTTAAT**ATG** |
| R1946 | CCCATCGGAGTTTTAAGGAGCTTAGAC**ATG** |
| R6925 | CCCATCGGAGTTTTAAGGAGGTTAGAC**ATG** |
| R21327 | CCCATCGGAGTTTTAAGGAGGTTTGAT**ATG** |
| R119539 | CCCATCGGAGTTTTAAGGAGGTTTAAT**ATG** |

*The web-interface RBS online calculator can be obtained from <https://salislab.net/>

**Table S8. Genotype and phenotypes of RBS optimized strains**

| Strains | 4.5-hour cultivation with or w/o ATC induction | OD_600_ | CFU/ml | Sequence mutation |
| --- | --- | --- | --- | --- |
| CIBT4523 | w/o | 5.42±0.02 | 4.0±1.2×10^8^ | N/A |
|  | w | 5.18±0.04 | 1.8±0.5×10^6^ | N/D |
| R1946 | w/o | 5.26±0.02 | 1.0±0.2×10^9^ | N/A |
|  | w | 1.31±0.04 | 4.85±1.55×10^5^ | Isolated colony 1：*dacA* 238_240del  Isolated colony 2：*dacA* 566C>T |
| R6925 | w/o | 5.25±0 | 1.0±0.2×10^9^ | N/A |
|  | w | 1.14±0.01 | 1.52±0.64×10^6^ | Isolated colony 1：*dacA* 155_162del  Isolated colony 2：mutation within Ptet |
| R21327 | w/o | 5.24±0.05 | 1.0±0.3×10^9^ | N/A |
|  | w | 3.29±0.33 | 4.60±3.40×10^6^ | Isolated colony 1：wt  Isolated colony 2：*tetR* 412G>A |
| R119539 | w/o | 5.19±0.07 | 1.0±0.2×10^9^ | N/A |
|  | w | 1.33±0.14 | 4.04±3.97×10^6^ | Isolated colony 1：mutation within Ptet  Isolated colony 2：*dacA* 488_489ins |
| R300 | w/o | 5.04±0.31 | 5.0±1.0×10^8^ | N/A |
|  | w | 4.57±0.06 | 1.7±0.4×10^8^ | N/A |
| R725 | w/o | 5.25±0.08 | 5.5±1.5×10^8^ | N/A |
|  | w | 2.22±0.03 | 6±1×10^4^ | N/A |
| R1099 (CIBT4707) | w/o | 5.19±0.02 | 7.7±2.4×10^7^ | N/A |
|  | w | 4.85±0.02 | 9±1×10^6^ | N/A |
| R1637 | w/o | 5.11±0 | 2.5±1.6×10^6^ | N/A |
|  | w | 1.40±0.12 | 2.4±0.3×10^5^ | N/A |

**Table S9. CFUs of RBS library on plates with different ampicillin concentrations**

| **Ampicillin concentration** μg/ml | CFUs per ml from host S | | CFUs per ml from W host | |
| --- | --- | --- | --- | --- |
|  | w/o ATC induction | With ATC induction | w/o ATC induction | With ATC induction |
| 12.5 | > 1000 | > 1000 | > 1000 | > 1000 |
| 25 | > 1000 | > 1000 | 98 | 60 |
| 50 | 10^3^ | 10^4^ | 3 | 11 |
| 100 | 1 | 1 | 1 | 5 |
| 200 | 0 | 1 | 0 | 0 |
| 400 | 0 | 0 | 0 | 0 |

| Strains selected from RBS library by ampicillin* | Host | Ampicillin concentration ug/ml | TIR sequence  (5’**NNNNNN** ATGGA**Y**TT**Y**3’) | *dacA* mutation |
| --- | --- | --- | --- | --- |
| TIR1 | S | 200 | TATTTGATGGATTTC（same as CIBT4523） | 402_765del |
| TIR2 | S | 100 | GGTACAATGGATTTT | wt |
| TIR3 | S | 100 | GCTACAATGGATTTT | wt |
| TIR4,21,29 | S | 50 | TATTTGATGGATTTC（same as CIBT4523） | wt |
| TIR5 | S | 50 | ATAATGATGGATTTT | 395T>A |
| TIR6,22,24,25,26,27,28 | S | 50 | CCCAATATGGATTTT | wt |
| TIR11,12,13, | W | 100 | GTGTTGATGGATTTC | 779del |
| TIR16,17,18,31,32,33,36 | W | 50 | GTGTTGATGGATTTC | 779del |
| TIR23 | S | 50 | GACGATAGTTCATAGGGTCCAGGCGG | 1_3del |
| TIR30 | S | 50 | CCGTGAATGGTTCAGTAACATGTCAATCTTGC | 1_3del |
| TIR34,35 | W | 50 | GCTAGAATGGATTTT | 810del |

**Table S10. TIR and dacA mutation in RBS library selected by ampicillin**

* Strains indicated in red were further selected for CDA production detection.

**Table S11. CDA production from selected strains**

| Strains | With or w/o ATC induction | OD600 | CFU/ml | CDA  (μM) |
| --- | --- | --- | --- | --- |
|  |  |  |  |  |
| CIBT4503 | w/o | 5.58 | 3.1×10^8^ | 0.00 |
|  | w | 5.63 | 6.6×10^7^ | 0.00 |
| CIBT4523 | w/o | 5.50 | 1.5×10^8^ | 0.89 |
|  | w | 5.51 | 4.2×10^7^ | 28.64 |
| Host S | w/o | 5.24 | 1.0×10^9^ | 0.16 |
|  | w | 5.23 | 1.0×10^9^ | 0.00 |
| Host W | w/o | 5.44 | 1.0×10^9^ | 0.21 |
|  | w | 5.49 | 1.0×10^9^ | 0.17 |
| TIR2 | w/o | 5.61 | 8.0×10^8^ | 0.00 |
|  | w | 4.30 | 1.5×10^8^ | 64.39 |
| TIR3.1 | w/o | 5.55 | 1.0×10^9^ | 0.00 |
|  | w | 4.28 | 1.0×10^7^ | 63.09 |
| TIR3.2 | w/o | 5.56 | 2.0×10^8^ | 0.00 |
|  | w | 4.27 | 5.0×10^7^ | 5.09 |
| TIR5.1 | w/o | 5.15 | 2.0×10^9^ | 0.81 |
|  | w | 5.38 | 2.0×10^9^ | 0.60 |
| TIR5.2 | w/o | 5.34 | 2.0×10^9^ | 1.30 |
|  | w | 4.05 | 6.8×10^7^ | 4.18 |
| TIR25 | w/o | 5.51 | 2.0×10^8^ | 0.50 |
|  | w | 4.04 | 2.0×10^6^ | 54.18 |
| TIR27 | w/o | 5.57 | 5.0×10^8^ | 1.78 |
|  | w | 4.29 | 6.0×10^6^ | 5.28 |
| TIR11 | w/o | 5.54 | 1.2×10^7^ | 0.17 |
|  | w | 5.50 | 1.0×10^8^ | 0.76 |
| TIR16 | w/o | 5.56 | 8.0×10^8^ | 0.20 |
|  | w | 5.52 | 2.0×10^8^ | 0.94 |
| TIR34 | w/o | 5.64 | 5.0×10^8^ | 0.00 |
|  | w | 5.61 | 1.5×10^8^ | 6.20 |
| TIR35 | w/o | 5.49 | 8.0×10^8^ | 0.19 |
|  | w | 5.54 | 8.0×10^8^ | 1.22 |

**Table S12. Strains for CDA production**

| Strains | With or w/o ATC induction | OD600 | CFU/ml | CDA  (μM) |
| --- | --- | --- | --- | --- |
|  |  |  |  |  |
| CIBT4503 | w/o | 5.30 | 1.0×10^9^ | 0.00 |
|  | w | 5.55 | 1.0×10^9^ | 0.00 |
| CIBT4523 | w/o | 5.34 | 1.0×10^9^ | 0.51 |
|  | w | 5.18 | 1.0×10^9^ | 24.02 |
| 4523Samp | w/o | 5.11 | 1.0×10^9^ | 0.00 |
|  | w | 5.26 | 1.0×10^9^ | 9.17 |
| 4523Wamp | w/o | 5.50 | 1.0×10^9^ | 0.00 |
|  | w | 5.18 | 1.0×10^9^ | 10.11 |
| TIR2 | w/o | 5.42 | 1.0×10^9^ | 0.31 |
|  | w | 4.03 | 6.0×10^7^ | 59.70 |
| TIR2ΔampR-1 | w/o | 4.92 | 1.0×10^9^ | 0.00 |
|  | w | 0.56 | 2.0×10^5^ | 1.74 |
| TIR2ΔampR-2 | w/o | 4.13 | 1.0×10^9^ | 0.00 |
|  | w | 1.57 | 1.1×10^6^ | 4.82 |
| TIR3.1ΔampR-1 | w/o | 5.27 | 1.0×10^9^ | 0.00 |
|  | w | 1.69 | 3.0×10^5^ | 18.49 |
| TIR3.1ΔampR-2 | w/o | 4.73 | 1.0×10^9^ | 0.00 |
|  | w | 1.55 | 5.0×10^5^ | 22.06 |
| TIR3.1ΔampR-1 | w/o | 5.27 | 1.0×10^9^ | 0.00 |
|  | w | 1.69 | 3.0×10^5^ | 18.49 |
| TIR25 | w/o | 5.07 | 1.0×10^9^ | 0.34 |
|  | w | 3.75 | 1.7×10^7^ | 46.75 |
| TIR25ΔampR-1 | w/o | 3.37 | 1.0×10^9^ | 0.35 |
|  | w | 0.31 | 5.6×10^6^ | 4.69 |
| TIR25ΔampR-2 | w/o | 4.96 | 1.0×10^9^ | 1.39 |
|  | w | 0.93 | 2.2×10^6^ | 9.03 |

**Table S13. Strains and plasmids**

| Strains and plasmids | Characteristics* | Source/Reference |
| --- | --- | --- |
| **Strains** |  |  |
| *E. coli* DH5α | *F- eNDA1 glnV44 thi-1 recA1 relA1 gyrA96 deoR nupG Φ80dlacZΔM15 Δ*(*lacZYA-argF*)*U169*, *hsdR17* (*rK-mK*), *λ–* | Takara |
| TransT1 | Trans1-T1 phage  resistant  chemically competent cell | TransGen |
| EcN | *E. coli* Nissle 1917 | Ardeypharm |
| CIBT8783 | EcN harboring plasmid pMW119K-Ptet-dacAec | This study |
| CIBT8784 | EcN harboring plasmid pMW119K-Ptet-dncVec | This study |
| CIBT8785 | EcN harboring plasmid pMW119K-Ptet-wspRec | This study |
| CIBT4502 | EcNΔ*dapA* | This study |
| CIBT4503 | EcNΔ*dapA*Δ*thyA* | This study |
| lacZ::dacAec1-6 | 6 transformants of EcNΔ*dapA*Δ*thyA*Δ*lacZ*::*dacA*ec | This study |
| CIBT4523 | CIBT4503 *exo/cea*::*dacA*lp | This study |
| CIBT8786 | CIBT4503 harboring plasmid pMB1S-dacAlp | This study |
| CIBT8787 | CIBT4503 harboring plasmid pSC101K-dacAlp | This study |
| CIBT4524 | CIBT4523Δ*lacZ*::Ptet-*dacA*lp | This study |
| CIBT8788 | CIBT4524Δ*yics*::*dacA*lp | This study |
| R300 | RBS of *dacA* in CIBT4523 was replaced with RBS300 sequence indicated in Table S6 | This study |
| R725 | RBS of *dacA* in CIBT4523 was replaced with RBS725 sequence indicated in Table S6 | This study |
| CIBT4707 | RBS of *dacA* in CIBT4523 was replaced with RBS1099 sequence indicated in Table S6 | This study |
| R1637 | RBS of *dacA* in CIBT4523 was replaced with RBS1637 sequence indicated in Table S6 | This study |
| R1946 | RBS of *dacA* in CIBT4523 was replaced with RBS1946 sequence indicated in Table S6 | This study |
| R6925 | RBS of *dacA* in CIBT4523 was replaced with RBS6925 sequence indicated in Table S6 | This study |
| R21327 | RBS of *dacA* in CIBT4523 was replaced with RBS21327 sequence indicated in Table S6 | This study |
| R119539 | RBS of *dacA* in CIBT4523 was replaced with RBS119539 sequence indicated in Table S6 | This study |
| 4523Samp | 4523Φ(*dacA-bla*), with strong coupling RBS sequence (引用） | This study |
| 4523Wamp | 4523Φ(*dacA-bla*), with weak coupling RBS sequence （引用） | This study |
| Host S | 4523Samp inserted with PAM sequences of Cas9 between Ptet and *dacA* | This study |
| Host W | 4523Wamp inserted with PAM sequences of Cas9 between Ptet and *dacA* | This study |
| TIR strains | TIR region within RBS of *dacA* in Host S or W was replaced with corresponding TIR sequence as indicated in Table S9, and *dacA* was mutated as indicated in Table S9 | This study |
| TIR2ΔampR | TIR2Δ*bla* | This study |
| TIR3ΔampR | TIR3Δ*bla* | This study |
| TIR25ΔampR | TIR25Δ*bla* | This study |
| TIR2ampR*（CIBT4712） | TIR2 with *bla* 22_44delinsTAATAA | This study |
| TIR3ampR* | TIR3 with *bla* 22_44delinsTAATAA | This study |
| TIR25ampR* | TIR25 with *bla* 22_44delinsTAATAA | This study |
| **Plasmids** | | |
| pMW119 | E. coli cloning vector; pSC101 oriV_E. coli._ *bla* lacZ | WAKO |
| pMW119K | Derived from pMW119 with ampR replaced with knR | This study |
| pLCNICK | E. coli-L. casei shuttle vector, repA101(Ts) repE Kn^r^ Cas9D10A P_ldh_-sgRNA | ^1^ |
| pMW119K-Ptet-dacAec | plasmid derived from pMW119K expressing *dacA*ec under control of Ptet | This study |
| pMW119K-Ptet-dncVec | plasmid derived from pMW119K expressing *dncV* from *Kingella denitrificans* ATCC 33394 (*E. coli* condon optimized) under control of Ptet | This study |
| pMW119K-Ptet-wspRec | plasmid derived from pMW119K expressing *wspR* from *Pseudomonas aeruginosa* PAO1 (*E. coli* condon optimized) under control of Ptet | This study |
| pCas | *repA101ts*, *kan^r^*, *P_cas_-cas9*, *P_araB_-Red*, *lacIq*, *P_trc_-*sgRNA-pMB1 | ^2^ |
| pTargetF | *pMB1 aadA* | ^2^ |
| pTargetF-dapA | *pMB1 aadA* sgRNA-*dapA* | This study |
| pTargetF-thyA | *pMB1 aadA* sgRNA-*thyA* | This study |
| pTargetF-lacZ | *pMB1 aadA* sgRNA-*lacZ* | This study |
| pTargetT-ΔlacZ::Ptet-dacAec | *pMB1 aadA* sgRNA-*lacZ* Δ*lacZ*:: Ptet-*dacA*ec | This study |
| pTargetF-exo/cea | *pMB1 aadA* sgRNA-*exo/cea* | This study |
| pTargetT-exo/cea::Ptet-dacAlp | *pMB1 aadA* sgRNA-*exo/cea exo/cea*::Ptet-*dacA*lp | This study |
| pMB1S-dacAlp | pMB1S derived plasmid expressing *dacA*lp under control of Ptet | This study |
| pKD46 | *repA101*(Ts) *bla araC* ParaB*-Red* | ^3^ |
| pSC101K-dacAlp | pSC101K derived plasmid expressing *dacA*lp under control of Ptet | This study |
| pTargetT-ΔlacZ::Ptet-dacAlp | *pMB1 aadA* sgRNA-*lacZ* Δ*lacZ*:: Ptet*-dacA*lp | This study |
| pTargetF-yics | *pMB1 aadA* sgRNA-*yics* | This study |
| pTargetT-yics::dacAlp | *pMB1 aadA* sgRNA-*yics yic*s::Ptet-*dacA*lp | This study |
| pTargetT-exo/cea::Ptet-dacAlp (R series) | *pMB1 aadA* sgRNA-*exo/cea* Δ*exo/cea*::Ptet-*dacA*lp, with corresponding RBS sequences indicated in Table S6 | This study |
| pTargetT-exo/cea::Ptet-dacAlp-SD(S)-AmpR | *pMB1 aadA* sgRNA-*exo/cea* Δ*exo/cea*::Ptet-Φ(*dacA-bla*), with strong coupling RBS sequence between *dacA* and *bla* ^4,5^ | This study |
| pTargetT-exo/cea::Ptet-dacAlp-SD(W)-AmpR | *pMB1 aadA* sgRNA-*exo/cea* Δ*exo/cea*::Ptet-Φ(*dacA-bla*), with weak coupling RBS sequence between *dacA* and *bla* ^4,5^ | This study |
| pTargetT-exo/cea::Ptet-PAM-dacAlp-SD(S)-AmpR | *pMB1 aadA* sgRNA-*exo/cea* Δ*exo/cea*::Ptet-Φ(PAM-*dacA-bla*), with strong coupling RBS sequence between *dacA* and *bla* (引用） | This study |
| pTargetT-exo/cea::Ptet-PAM-dacAlp-SD(W)-AmpR | *pMB1 aadA* sgRNA-*exo/cea* Δ*exo/cea*::Ptet-Φ(PAM-*dacA-bla*), with weak coupling RBS sequence between *dacA* and *bla* ^4,5^ | This study |
| pTargetF-RBSdacA | *pMB1 aadA* sgRNA- RBS (PAM before *dacA*) | This study |
| pTargetT-TIR | *pMB1 aadA* sgRNA- RBS (PAM before *dacA*)，Δ*dacA*::TIR | This study |
| pUC57 | pUC19-derived prokaryotic expression vector | GeneScript |
| pTargetF-AmpR | *pMB1 aadA* sgRNA-*bla* | This study |
|  |  |  |

*bla*, ampicillin resistance gene; *kn^r^*, kanamycin resistance gene; *aadA*, spectinomycin resistance gene; TIR, translation initiation region; *dacA*lp, *dacA* from *Listeria monocytogen* with *Lactobacillus plantarum* codon optimized; *dacA*ec, *dacA* from *Listeria monocytogen* with *E. coli* codon optimized; PAM, the protospacer-adjacent motif; Ptet, tetracycline-responsive promoter; sgRNA*-x,* sgRNA with an N20 sequence for targeting the *x* locus; Δ*y*::z, editing template with homologous fragment to the *y* locus with a *z* fragment insertion.

**Table S14. Primers used in the study**

| Primers | Sequences (5’-3’) | **Characteristics** |
| --- | --- | --- |
| 119-F | aggccctttcgtcttcaagaa | to clone pMW119K |
| 119-R | gtgcacacagcccagcttgga |  |
| 119-kan-F | cggtgtaggtcgttcgctccaagctgggctgtgtgcacatccttttaaattaaaaatga |  |
| 119-kan-R | acccgtcttactgtcaattcttgaagacgaaagggcctctgcagggaaagccacgttgt |  |
| 119-tetR-F | ttcccagtcacgacgttgtaaaacgacggccagtgaattcctgatgaatcccctaatga | to clone pMW119K-Ptet-dacAec |
| pTet-R | atgtatatctccttcttaaag |  |
| ptet-dacA-F | tagaaataattttgtttaactttaagaaggagatatacatatggacttcagcaacatga |  |
| rrnB-dacA-R | agttccctactctcgcatggggagaccccacactaccatcttactcgcttttgccacct |  |
| rrnBT1T2-F | gatggtagtgtggggtct |  |
| 119-rrnBT1T2-R | cgccaagcttgcatgcctgcaggtcgactctagaggatccaaaaggccatccgtcagga |  |
| ptet-R1 | atgtatatctccttcttaaagttaaacaaaattatttc | to clone pMW119K-Ptet-dncVec |
| ptet-DncV-Kd(ZL)-F | gaaataattttgtttaactttaagaaggagatatacatatgtcagactacactaataac |  |
| rrnB-DncV-Kd(ZL)-R | ttccctactctcgcatggggagaccccacactaccatcttaaccctggcgcaagggcac |  |
| rrnB-DncV-Ve(ZL)-R | ccctactctcgcatggggagaccccacactaccatcttagcgacggcggcggaataaac |  |
| ptet-WspR-pae(EC)-F | gaaataattttgtttaactttaagaaggagatatacatatgcacaacccgcacgaaagc | to clone pMW119K-Ptet-wspRec |
| rrnB-WspR-pae(EC)-R | cctactctcgcatggggagaccccacactaccatcttaacccgccggcgccggcggcac |  |
| WspR(D70E)-R | tcagaccatcaacacccggcatcaccagttcctgcagaataacggtcggtttgatttgg |  |
| N20-dapA-F | tcctaggtataatactagtagatcgcagccagtacgggagttttagagctagaaatagc | to clone pTargetF-dapA |
| N20-R | actagtattatacctaggac |  |
| N20-thyA-F | tcctaggtataatactagtaccggaacgctttccattttgttttagagctagaaatagc | to clone pTargetF-thyA paired with primer N20R |
| N20-lacZ-F | tcctaggtataatactagtggcaagcggtgaagtgcctcgttttagagctagaaatagc | to clone pTargetF-lacZ paired with primier N20-R |
| lacZ-L-F1 | cttgaaaaagtggcaccgagtcggtgctttttttgaattctacggggtatacatgtctg | to clone pTargetT-ΔlacZ::Ptet-dacAec |
| lacZ-L-R2 | gggaaaaccctggcgttacccaacttaatcgccttgcagccacatcagcgcctggcag |  |
| tetR-ptet-dacA-F2 | tgctgccaggcgctgatgtggctgcaaggcgattaagttg |  |
| tetR-ptet-dacA-R2 | ctggcaggcgtttcgtcagtaacacaggaaacagctatgac |  |
| lacZ-R-F2 | aagcttggcgtaatcatggtcatagctgtttcctgtgttactgacgaaacgcctgccag |  |
| lacZ-R-R1 | taatagatctaagcttctgcaggtcgactctagagaattctggtctgctgctgctgaac |  |
| N20-exo-F | tcctaggtataatactagttttattgatatatttacgtcgttttagagctagaaatagc | to clone pTargetF-exo/cea paired with primer N20-R |
| exo-L-F | cttgaaaaagtggcaccgagtcggtgctttttttgaattcgcttccagaggaagctttg | to clone pTargetT-exo/cea::Ptet-dacAlp |
| tetR-exo-L-R | tggcgttacccaacttaatcgccttgcagcctggataacgtaaatgattg |  |
| exoL-tetR-F | tgtataccgcaatcatttacgttatccaggctgcaaggcgattaagttgg |  |
| RBS385-ptet-R | caaatagtggacctcctttaaagttaaacaaaattatttc |  |
| Ptet-dacA(LcLp)-F | aaataattttgtttaactttaaaggaggtccactatttgatggatttcagtaacatgtc |  |
| rrnB-dacA(LcLp)-R | tccctactctcgcatggggagaccccacactaccatcttattctgacttgccacccttc |  |
| rrnBT1T2-F | gatggtagtgtggggtct |  |
| exoR-rrnB-R | ctacattaggtatctgggggattgctattacacaggaaacagctatgacc |  |
| rrnB-exoR-F | cgtaatcatggtcatagctgtttcctgtgtaatagcaatcccccagatac |  |
| exo-R-R | atgaactcgagtagggataacagggtaatagatctaagctttgtccggctcagttaacc |  |
| pMB1-tetR-F | cgcatctgtgcggtatttcacaccgcatatgctggatccgctgcaaggcgattaagttg | to clone pMB1S-dacAlp |
| rrnBT1T2-R-R | atgaactcgagtagggataacagggtaatagatctaagcttacacaggaaacagctatg |  |
| Kan-pSC101-F | agtttttctaatcagaattggttaattggttgctgcagtgggttgatgataccgctgcc | to clone pSC101K-dacAlp |
| tetR-repA-R | aaaaccctggcgttacccaacttaatcgccttgcagccatatggacagttttccctttg |  |
| tetR-F | gctgcaaggcgattaagttg |  |
| tetR-dacA(LcLp)-R | acacaggaaacagctatgacc |  |
| rrnB-kan-F1 | cttggcgtaatcatggtcatagctgtttcctgtgtttgttatcattctatagtattaag |  |
| pSC101-kan-R | catcaacccactgcagcaaccaattaaccaattctgattagaaaaactcatcgagcatc |  |
| N20-yics-F | tcctaggtataatactagtgatatcaggtaaagaagcgtgttttagagctagaaatagc | to clone pTargetF-yics paired with primer N20-R |
| yicS-L-F | ttgaaaaagtggcaccgagtcggtgctttttttgaattctcgcaacctgccagcagaac | to clone pTargetT-yics::dacAlp |
| tetR-yicsL-R | tggcgttacccaacttaatcgccttgcagcgtcagtggttgttgccgttc |  |
| rrnB-yicSR-F | taatcatggtcatagctgtttcctgtgtcaacgcttctttacctgatatc |  |
| yicS-R-R | gaactcgagtagggataacagggtaatagatctaagctttgaaccttcgcatcagaaac |  |
| dacA(LcLp)-F(R300) | gtttaactttaacccatcggagttttaggcagcttaaacatggatttcagtaacatgtc | to clone pTargetT-exo/cea::Ptet-dacAlp(R300) |
| dacA(LcLp)-R(R300) | atccatgtttaagctgcctaaaactccgatgggttaaagttaaacaaaattatttctag |  |
| dacA(LcLp)-F(R725) | gtttaactttaacccatcggagttttatggagctttaacatggatttcagtaacatgtc | to clone pTargetT-exo/cea::Ptet-dacAlp(R725) |
| dacA(LcLp)-R(R725) | atccatgttaaagctccataaaactccgatgggttaaagttaaacaaaattatttctag |  |
| dacA(LcLp)-F(R1099) | gtttaactttaacccatcggagttttaaggagctttgatatggatttcagtaacatgtc | to clone pTargetT-exo/cea::Ptet-dacAlp(R1099) |
| dacA(LcLp)-R(R1099) | atccatatcaaagctccttaaaactccgatgggttaaagttaaacaaaattatttctag |  |
| dacA(LcLp)-F(R1637) | gtttaactttaacccatcggagttttaggcaggtttaatatggatttcagtaacatgtc | to clone pTargetT-exo/cea::Ptet-dacAlp(R1637) |
| dacA(LcLp)-R(R1637) | atccatattaaacctgcctaaaactccgatgggttaaagttaaacaaaattatttctag |  |
| dacA(LcLp)-F(R1946) | gtttaactttaacccatcggagttttaaggagcttagacatggatttcagtaacatgtc | to clone pTargetT-exo/cea::Ptet-dacAlp(R1946) |
| dacA(LcLp)-R(R1946) | atccatgtctaagctccttaaaactccgatgggttaaagttaaacaaaattatttctag |  |
| dacA(LcLp)-F(R6925) | gtttaactttaacccatcggagttttaaggaggttagacatggatttcagtaacatgtc | to clone pTargetT-exo/cea::Ptet-dacAlp(R6925) |
| dacA(LcLp)-R(R6925) | atccatgtctaacctccttaaaactccgatgggttaaagttaaacaaaattatttctag |  |
| dacA(LcLp)-F(R21327) | gtttaactttaacccatcggagttttaaggaggtttgatatggatttcagtaacatgtc | to clone pTargetT-exo/cea::Ptet-dacAlp(R21327) |
| dacA(LcLp)-R(R21327) | atccatatcaaacctccttaaaactccgatgggttaaagttaaacaaaattatttctag |  |
| dacA(LcLp)-F(R119539) | gtttaactttaacccatcggagttttaaggaggtttaatatggatttcagtaacatgtc | to clone pTargetT-exo/cea::Ptet-dacAlp(R119539) |
| dacA(LcLp)-R(R119539) | atccatattaaacctccttaaaactccgatgggttaaagttaaacaaaattatttctag |  |
| HindIII-F(N20) | aagcttagatctattaccctg |  |
| dacA(LcLp)-SD(S)-Amp-F | ggtggcaagtcagaatgacataggaggtcctcctatgtcaattcaacatttccgtgtcg | to clone pTargetT-exo/cea::Ptet-dacAlp-SD(S)-AmpR |
| rrnBT1T2-Amp-R | cagttccctactctcgcatggggagaccccacactaccatcttaccaatgcttaatcag |  |
| dacA(LcLp)(TGA)-R | tcattctgacttgccacccttcc |  |
| dacA(LcLp)-SD(W)-Amp-F | ggtggcaagtcagaatgaaataggaggtcctcctatttcaattcaacatttccgtgtcg | to clone pTargetT-exo/cea::Ptet-dacAlp-SD(W)-AmpR |
| ptet-RBS(N20)-dacA(LcLp)-F | ttaaaggaggtccacgacgatagttcatagggtccaggcggagtaacatgtcaatcttg | to clone pTargetT-exo/cea::Ptet-PAM-dacAlp-SD(S)-AmpR-RBS-N20 or  pTargetT-exo/cea::Ptet-PAM-dacAlp-SD(W)-AmpR |
| dacA(LcLp)-RBS(N20)-ptet-R | acatgttactccgcctggaccctatgaactatcgtcgtggacctcctttaaagttaaac |  |
| dacAlp-RBS(N20)-F | tcctaggtataatactagtgacgatagttcatagggtccgttttagagctagaaatagc | To clone pTargetF-RBSdacA paired with primer N20-R |
| tetR-F(EcoRI) | aaaagtggcaccgagtcggtgctttttttgaattcttaagacccactttcacatttaag | To clone pTargetT-TIR |
| ptet-R | gtggacctcctttaaagttaaac |  |
| dacA(LcLp)-F | agtaacatgtcaatcttgc |  |
| dacA(LcLp)-R(HindIII) | atgaactcgagtagggataacagggtaatagatctaagctttcattctgacttgccacc |  |
| ptet-TIR-dacA(LcLp)-F1 | tttaactttaaaggaggtccacnnnnnnatggayttyagtaacatgtcaatcttgcatt |  |
| dacA(LcLp)-TIR-ptet-R | aatgcaagattgacatgttactraartccatnnnnnngtggacctcctttaaagttaaa |  |
| N20-AmpR-F | tcctaggtataatactagtaaaagggaataagggcgacagttttagagctagaaatagc | To clone pTargetF-AmpR paired with primer N20-R |
| dapA-F | gacttttgaacagagtaagccatcaaatctccctaaactgggccatcctctgtgcaaac | To generate donor DNA deleting *dapA* |
| dapA-R | tgcttttaatgccataccaaacgtaccattgagacacttgtttgcacagaggatggccc |  |
| thyA-F | tatcgtcgcagcccacagcaacacgtttcctgaggaacctggaccggtggcgacacgca | To generate donor DNA deleting *thyA* |
| thyA-R | aattgcagatgagtttgatccatatggttgctgtagagatgcgtgtcgccaccggtcca |  |
| AmpR-F | aagtatttttagtaagtggaagggtggcaagtcagaatgagatggtagtgtggggtctc | To generate donor DNA deleting *ampR* |
| AmpR-R | tccctactctcgcatggggagaccccacactaccatctcattctgacttgccacccttc |  |
| AmpR(TAA)-F | aggaggtcctcctatgtcaattcaacatttccgttaataattgcggcattttgccttcc | To generate donor DNA silent mutating *ampR* with *bla* 22_44delinsTAATAA |
| AmpR(TAA)-R | gggtgagcaaaaacaggaaggcaaaatgccgcaattattaacggaaatgttgaattgac |  |
| tetR-seq-F | agtgagtatggtgcctatct | Targeting *tetR* locus |
| 119-seq-F | aaatcatggcaattctgg | Targeting pMW119K series plasmid |
| 119-seq-R | cattaatgcagctggcacga |  |
| 119-kan-seq-R | tctgctatgtggtgctatct | Targeting *kanR* in pMW119K series |
| N20-seq-F | gctcacatgttctttcctgc | Targeting N20 locus in pTargetF series |
| lacZ-L-seq-F | tcgccaatccacatctgtg | Targeting *lacZ* locus |
| lacZ-R-seq-R | cgatgggtaacagtcttgg |  |
| exo-L-seq-F | acactcagcgatcgctgacg | Targeting *exo* locus |
| exo-R-seq-R | gctaaatggtctactgctc |  |
| N20-seq-R | gacattgcactccaccgctg | Targeting pMB1S-dacAlp |
| repA-seq-F | gtaagcatgatctcaatgg | Targeting pSC101K-dacAlp |
| kan-seq-R | tgtttcagaaacaactctgg |  |
| yics-L-seq-F | gcgtcattatcagatgaagc | Targeting *yics* locus |
| yics-R-seq-R | cgttggataacattggtctgac |  |
| dacA(LcLp)-seq-F | gttgatcaacatcttcatcc | Targeting SD sequence before *ampR* |
| dapA-seq-F | gttccattgtacccaatcg | Targeting *dapA* locus |
| dapA-seq-R | acttgttctccgtcttgatcc |  |
| thyA-seq-F | aatgtggctggcaacacgac | Targeting *thyA* locus |
| thyA-seq-R | ttctccttgctgacaaagc |  |
| lacZ-seq-F | actgacgggctccaggag | Targeting *lacZ* locus |
| lacZ-seq-R | ggtgaaattatcgatgagcg |  |
| exo-seq-F | atgacctcctgctctgcac | Targeting *exo* locus |
| exo-seq-R | tgtgctaccggaacgactc |  |
| yicS-seq-F | tcaagaaagggtcgatgctgg | Targeting *yjcS* locus |
| yicS-seq-R | ctgaccatgcgtctggtacc |  |

**Table S15. Materials and methods used for plasmids cloning in this study**

| Plasmid | Fragment | Upstream primer or restriction enzyme used | Downstream primer or restriction enzyme used | Template for PCR or restriction enzyme digestion | Tartgeted fragment length（bp） | Characteristics | Resistance | Cloing method^6^ |
| --- | --- | --- | --- | --- | --- | --- | --- | --- |
| pMW119K | D1 | 119-F | 119-R | pMW119 | 2660 | Plasmid backbone | Kan | Isothermal assemble of D1 and D2 |
|  | D2 | 119-kan-F | 119-kan-R | pLCNICK | 1109 | *kn^r^* |  |  |
| pMW119K-Ptet-dacAec | D3 | *EcoR I* | *BamH I* | pMW119K | 3676 | Plasmid backbone | Kan | Isothermal assemble of D3, D4, D5 and D6 |
|  | D4 | 119-tetR-F | ptet-R | Synthesized *tetR*-Ptet fragment | 857 | *tetR*-Ptet |  |  |
|  | D5 | ptet-dacA-F | rrnB-dacA-R | Synthesized *E. coli* codon optimized *dacA* | 822 | *dacA* |  |  |
|  | D6 | rrnBT1T2-F | 119-rrnBT1T2-R | *E. coli* MG1655 genome | 294 | *rrnBT1T2* |  |  |
| pMW119K-Ptet-dncVec | D7 | rrnBT1T2-F | ptet-R1 | pMW119K-Ptet-dacAec | 4755 | Plasmid backbone | Kan | Isothermal assemble of D7 and D8 |
|  | D8 | ptet-DncV-Kd(ZL)-F | rrnB-DncV-Kd(ZL)-R | Synthesized *E. coli* codon optimized *dnvV* | 1287 | *dncV* |  |  |
| pMW119K-Ptet-wspRec | D11 | ptet-WspR-pae(EC)-F | rrnB-WspR-pae(EC)-R | Synthesized *E. coli* codon optimized *wspR* | 1116 | *wspR* | Kan | Isothermal assemble of D7 and D11 |
| pTargetT-ΔlacZ::Ptet-dacAec | D18 | *EcoR I* | | pTargetF-lacZ | 2118 | Plasmid backbone | Spec | Isothermal assemble of D18, D19,  D20, and D21 |
|  | D19 | lacZ-L-F1 | lacZ-L-R2 | EcN genome | 650 | Upstream homologous fragment of *lacZ* |  |  |
|  | D20 | tetR-ptet-dacA-F2 | tetR-ptet-dacA-R2 | pMW119K-Ptet-dacAec | 2038 | Plasmid backbone |  |  |
|  | D21 | lacZ-R-F2 | lacZ-R-R1 | EcN genome | 660 | Downstream homologous fragment of *lacZ* |  |  |
| pTargetT-exo/cea::Ptet-dacAlp | D23 | *EcoR I* | *Hind III* | pTargetF-exo/cea | 2118 | Plasmid backbone | Spec | Isothermal assemble of D23, D24,  D28, and D29 |
|  | D24 | exo-L-F | tetR-exo-L-R | EcN genome | 623 | Upstream homologous fragment |  |  |
|  | D25 | exoL-tetR-F | RBS385-ptet-R | pMW119K-Ptet-dacAec | 895bp | *tetR*-Ptet-RBS fragment |  |  |
|  | D26 | Ptet-dacA(LcLp)-F | rrnB-dacA(LcLp)-R | Synthesized *L. plantarum* codon optimized *dacA* | 822 | *dacA* |  |  |
|  | D27 | rrnBT1T2-F | exoR-rrnB-R | pMW119K-Ptet-dacAec | 323 | *rrnBT1T2* |  |  |
|  | D28 | exoL-tetR-F | exoR-rrnB-R | D25，D26，D27 | 2040 | Overlap PCR to generate *tetR*-Ptet-RBS-*dacA- rrnBT1T2* fragment |  |  |
|  | D29 | rrnB-exoR-F | exo-R-R | EcN genome | 634 | Downstream homologous fragment |  |  |
| pMB1S-dacAlp | D30 | *BamH I* | *Hind III* | pTargetF | 1950 | Plasmid backbone | Spec | Isothermal assemble of D30 and D31 |
|  | D31 | pMB1-tetR-F | rrnBT1T2-R-R | pTargetT-exo/cea::Ptet-dacAlp | 2040 | *tetR*-Ptet-RBS-*dacA- rrnBT1T2* fragment |  |  |
| pSC101K-dacAlp | D32 | Kan-pSC101-F | tetR-repA-R | pKD46 | 1800 | Plasmid backbone | Kan | Isothermal assemble of D32, D33 and D34 |
|  | D33 | tetR-F | tetR-dacA(LcLp)-R | pTargetT-exo/cea::Ptet-dacAlp | 2040 | *tetR*-Ptet-RBS-*dacA- rrnBT1T2* fragment |  |  |
|  | D34 | rrnB-kan-F1 | pSC101-kan-R | pMW119K | 1000 | *kn^r^* |  |  |
| pTargetT-ΔlacZ::dacAlp | D35 | rrnBT1T2-F | RBS385-ptet-R | pTargetT-ΔlacZ::Ptet-dacAec | 4652 | Plasmid backbone | Spec | Isothermal assemble of D26, and D35 |
| pTargetT-yics::dacAlp | D37 | *EcoR I* | *Hind III* | pTargetF-yics | 2118 | Plasmid backbone | Spec | Isothermal assemble of D33, D37  D38, and D39 |
|  | D38 | yicS-L-F | tetR-yicsL-R | EcN genome | 609 | Upstream homologous fragment of *yics* |  |  |
|  | D39 | rrnB-yicSR-F | yicS-R-R | EcN genome | 612 | Downstream homologous fragment of *yics* |  |  |
| pTargetT-exo/cea::Ptet-dacAlp (R300) | D40 | dacA(LcLp)-F(R300) | exo-R-R | pTargetT-exo/cea::Ptet-dacAlp | 1800 | Plasmid backbone | Spec | Isothermal assemble of D40 and D41 |
|  | D41 | HindIII-F(N20) | dacA(LcLp)-R(R300) | pTargetT-exo/cea::Ptet-dacAlp | 3600 | Homologous fragment |  |  |
| pTargetT-exo/cea::Ptet-dacAlp (R725) | D42 | dacA(LcLp)-F(R725) | exo-R-R | pTargetT-exo/cea::Ptet-dacAlp | 1800 | Plasmid backbone | Spec  Spec | Isothermal assemble of D42 and D43 |
|  | D43 | HindIII-F(N20) | dacA(LcLp)-R(R725) | pTargetT-exo/cea::Ptet-dacAlp | 3600 | Homologous fragment |  |  |
| pTargetT-exo/cea::Ptet-dacAlp (R1099) | D44 | dacA(LcLp)-F(R1099) | exo-R-R | pTargetT-exo/cea::Ptet-dacAlp | 1800 | Plasmid backbone | Spec  Spec | Isothermal assemble of D44 and D45 |
|  | D45 | HindIII-F(N20) | dacA(LcLp)-R(R1099) | pTargetT-exo/cea::Ptet-dacAlp | 3600 | Homologous fragment |  |  |
| pTargetT-exo/cea::Ptet-dacAlp (R1637) | D46 | dacA(LcLp)-F(R1637) | exo-R-R | pTargetT-exo/cea::Ptet-dacAlp | 1800 | Plasmid backbone | Spec  Spec | Isothermal assemble of D46 and D47 |
|  | D47 | HindIII-F(N20) | dacA(LcLp)-R(R1637) | pTargetT-exo/cea::Ptet-dacAlp | 3600 | Homologous fragment |  |  |
| pTargetT-exo/cea::Ptet-dacAlp (R1946) | D48 | dacA(LcLp)-F(R1946) | exo-R-R | pTargetT-exo/cea::Ptet-dacAlp | 1800 | Plasmid backbone | Spec | Isothermal assemble of D48 and D49 |
|  | D49 | HindIII-F(N20) | dacA(LcLp)-R(R1946) | pTargetT-exo/cea::Ptet-dacAlp | 3600 | Homologous fragment |  |  |
| pTargetT-exo/cea::Ptet-dacAlp(R6925) | D50 | dacA(LcLp)-F(R6925) | exo-R-R | pTargetT-exo/cea::Ptet-dacAlp | 1800 | Plasmid backbone | Spec | Isothermal assemble of D50 and D51 |
|  | D51 | HindIII-F(N20) | dacA(LcLp)-R(R6925) | pTargetT-exo/cea::Ptet-dacAlp | 3600 | Homologous fragment |  |  |
| pTargetT-exo/cea::Ptet-dacAlp (R21327) | D52 | dacA(LcLp)-F(R21327) | exo-R-R | pTargetT-exo/cea::Ptet-dacAlp | 1800 | Plasmid backbone | Spec | Isothermal assemble of D52 and D53 |
|  | D53 | HindIII-F(N20) | dacA(LcLp)-R(R21327) | pTargetT-exo/cea::Ptet-dacAlp | 3600 | Homologous fragment |  |  |
| pTargetT-exo/cea::Ptet-dacAlp (R119539) | D54 | dacA(LcLp)-F(R119539) | exo-R-R | pTargetT-exo/cea::Ptet-dacAlp | 1800 | Plasmid backbone | Spec | Isothermal assemble of D54 and D55 |
|  | D55 | HindIII-F(N20) | dacA(LcLp)-R(R119539) | pTargetT-exo/cea::Ptet-dacAlp | 3600 | Homologous fragment |  |  |
| pTargetT-exo/cea::Ptet-dacAlp-SD(S)-AmpR | D56 | rrnBT1T2-F | dacA(LcLp)  (TGA)-R | pTargetT-exo/cea::Ptet-dacAlp | 5400 | Plasmid backbone | Spec | Isothermal assemble of D56 and D57 |
|  | D57 | dacA(LcLp)-SD(S)-Amp-F | rrnBT1T2-Amp-R | pUC57 | 861 | SD(S)-*bla* |  |  |
| pTargetT-exo/cea::Ptet-dacAlp-SD(W)-AmpR | D58 | dacA(LcLp)-SD(W)-Amp -F | rrnBT1T2-Amp-R | pUC57 | 861 | SD(W)-*bla* | Spec | Isothermal assemble of D56 and D58 |
| pTargetT-exo/cea::Ptet-PAM-dacAlp-SD(S)-AmpR | D59 | rrnBT1T2-F | dacA(LcLp)-RBS(N20)-ptet-R | pTargetT-exo/cea::Ptet-dacAlp | 4600 | Plasmid backbone | Spec | Isothermal assemble of D59 and D60 |
|  | D60 | ptet-RBS(N20)-dacA(LcLp)-F | rrnBT1T2-Amp-R | pTargetT-exo/cea-Ptet::dacAlp-SD(S)-AmpR | 1700 | PAM-dacAlp-SD(S)-*bla* fragment |  |  |
| pTargetT-exo/cea::Ptet-PAM-dacAlp-SD(W)-AmpR | D61 | rrnBT1T2-F | dacA(LcLp)-RBS(N20)-ptet-R | pTargetT-exo/cea-Ptet-dacAlp | 4600 | Plasmid backbone | Spec | Isothermal assemble of D61 and D62 |
|  | D62 | ptet-RBS(N20)-dacA(LcLp)-F | rrnBT1T2-Amp-R | pTargetT-exo/cea-Ptet-dacAlp-SD(W)-AmpR | 1700 | PAM-dacAlp-SD(w)-*bla* fragment |  |  |
| pTargetT-TIR library | D64 | *EcoR I* | *Hind III* | pTargetF-RBSdacA | 2118 | Plasmid backbone | Spec | Isothermal assemble of D67 and D68 |
|  | D65 | tetR-F(EcoRI) | ptet-R | pTargetT-exo/cea::Ptet-dacAlp | 750 | Upstream homologous fragment |  |  |
|  | D66 | dacA(LcLp)-F | dacA(LcLp)-R(HindIII) | pTargetT-exo/cea::Ptet-dacAlp | 830 | Downstream homologous fragment |  |  |
|  | D67 | dacA(LcLp)-F | ptet-R | D65, D64, D66 | 3700 | Overlap PCR to fuse D65, D64 and D66 |  |  |
|  | D68 | ptet-TIR-dacA(LcLp)-F1 | dacA(LcLp)-TIR-ptet-R | ptet-TIR-dacA(LcLp)-F1 and dacA(LcLp)-TIR-ptet-R | 59 | Primers annealing to generate double strand DNA fragment library |  |  |

**Table S16. Materials used in recombinent strains constructions by CRISPR/Cas method^2^**

| Final strains | Host | Plasmids | Donor DNA^#^ |
| --- | --- | --- | --- |
| CIBT4502 | EcN | pCas, pTargetF-dapA | ΔdapA generated by annealing primers dapA-Fand dapA-R |
| CIBT4503 | CIBT4502 | pCas, pTargetF-thyA | ΔthyA generated by annealing primersthyA-F and thyA-R |
| lacZ::dacAec | CIBT4503 | pCas, pTargetT-ΔlacZ::Ptet-dacAec |  |
| CIBT4523 | CIBT4503 | pCas, pTargetT-exo/cea::Ptet-dacAlp |  |
| CIBT4524 | CIBT4523 | pCas，pTargetT-ΔlacZ::dacAlp |  |
| CIBT8788 | CIBT4524 | pCas，pTargetT-yics::dacAlp |  |
| R series | CIBT4503 | pCas，pTargetT-exo/cea::Ptet-dacAlp (Rseries) |  |
| 4523Samp | CIBT4503 | pCas, pTargetT-exo/cea::Ptet-dacAlp-SD(S)-AmpR |  |
| 4523Wamp | CIBT4503 | pCas, pTargetT-exo/cea-Ptet::dacAlp-SD(W)-AmpR |  |
| Host S | CIBT4503 | PCas, pTargetT-exo/cea::Ptet-PAM-dacAlp-SD(S)-AmpR |  |
| Host W | CIBT4503 | pTargetT-exo/cea::Ptet-PAM-dacAlp-SD(W)-AmpR |  |
| TIRΔampR series | A corresponding TIR strain | pCas, pTargetF-AmpR | ΔAmpR generated by annealing primers AmpR-F and AmpR-R |
| TIRampR* series | A corresponding TIR strain | pCas, pTargetF-AmpR | ΔAmpRTAA generated by annealing primers AmpR-F(TAA)-F and AmpR(TAA)-R |

^#^The donor DNA were obtained by annealing relative primers (the annealing temperature decreases by 1 degree every 10 seconds.

**Supplementary Methods**

**Quantification of CDA in tumor tissues**

The B16F10 (1 × 10^5^) cells were subcutaneously injected into C57BL/6 mice. When the tumors reached a volume of 300–500 mm^3^, the mice were randomized into different groups, as indicated. The mice were intraperitoneally injected with 60 µL of ATC (200 µg/mL), and 4 h later, 100 µL of CIBT4523 (1 × 10^8^ CFUs), CIBT4503 (1 × 10^8^ CFUs), or vehicle (PBS) was administered. After 24 h, the mice were euthanized, and the tumors were removed via dissection. Harvested tumors were weighed and placed in sterile homogenizer tubes prefilled with 3.0 mm Grinding beads (Shanmi, YM-30), frozen and stored at −80 °C to allow for quantification of all samples and time points in the same run. Homogenized for 60s in utilizing a High-throughput tissue grinder (SCIENTZ, SCIENTZ-48). Tumor homogenate was extracted with 9 parts methanol, vortexed, and centrifuged at 2300g for 5 min at 4 °C. Supernatants were diluted 10-fold with 0.1% formic acid prior to analysis.

CDA was separated and detected on a DIONEX UltiMate 3000/ TSQ Quantiva LC-MS/MS system (Thermo Fisher Scientific). Samples were injected (1 μL) and separated on a ACQUITY UPLC HSS T3 (3.0 × 150 mm, 1.8 μm, Waters) and analyte eluted using a linear gradient of 8 to 95% B mixed with A from2 to 6 min (A: 20mM ammonium acetate ; B: Methanol) at 0.3 mL/min at 35 °C. CDA was detected using selected reaction monitoring (SRM) of compound specific mass transition 659.1 > 312， 659.1 > 330，659.1 > 524 m/z in positive electrospray ionization mode. Peaks were integrated and peak areas used to calculate concentrations of the unknowns.

**Supplemental Figures**


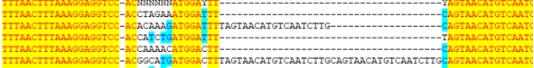


**Supplemental Figure 1.** RBS sequences in RBS library. The randomized sequences of translation initiation region were indicated in line 1, and the random picked 5 strains were sequenced and blasted as indicated in the following lines.


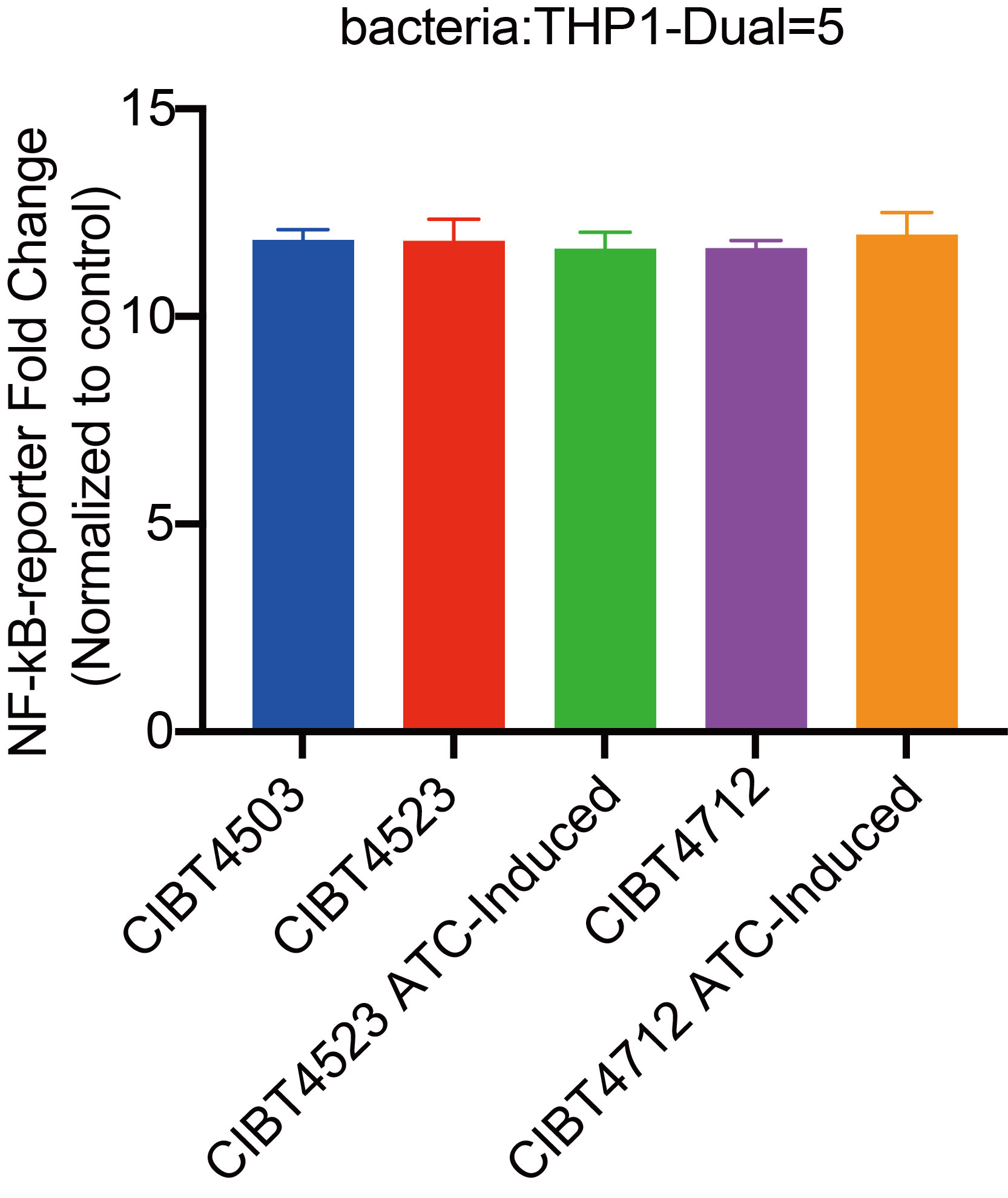


**Supplemental Figure 2** Activity of engineered strains on NF-κB pathway. The engineered strains with or without ATC (200 μg/ml) induction were added to THP1-Dual cells for 24 h, the fold change of NF-κB reporter was calculated relative to control. No significant difference was found among different groups using One-way ANOVA.


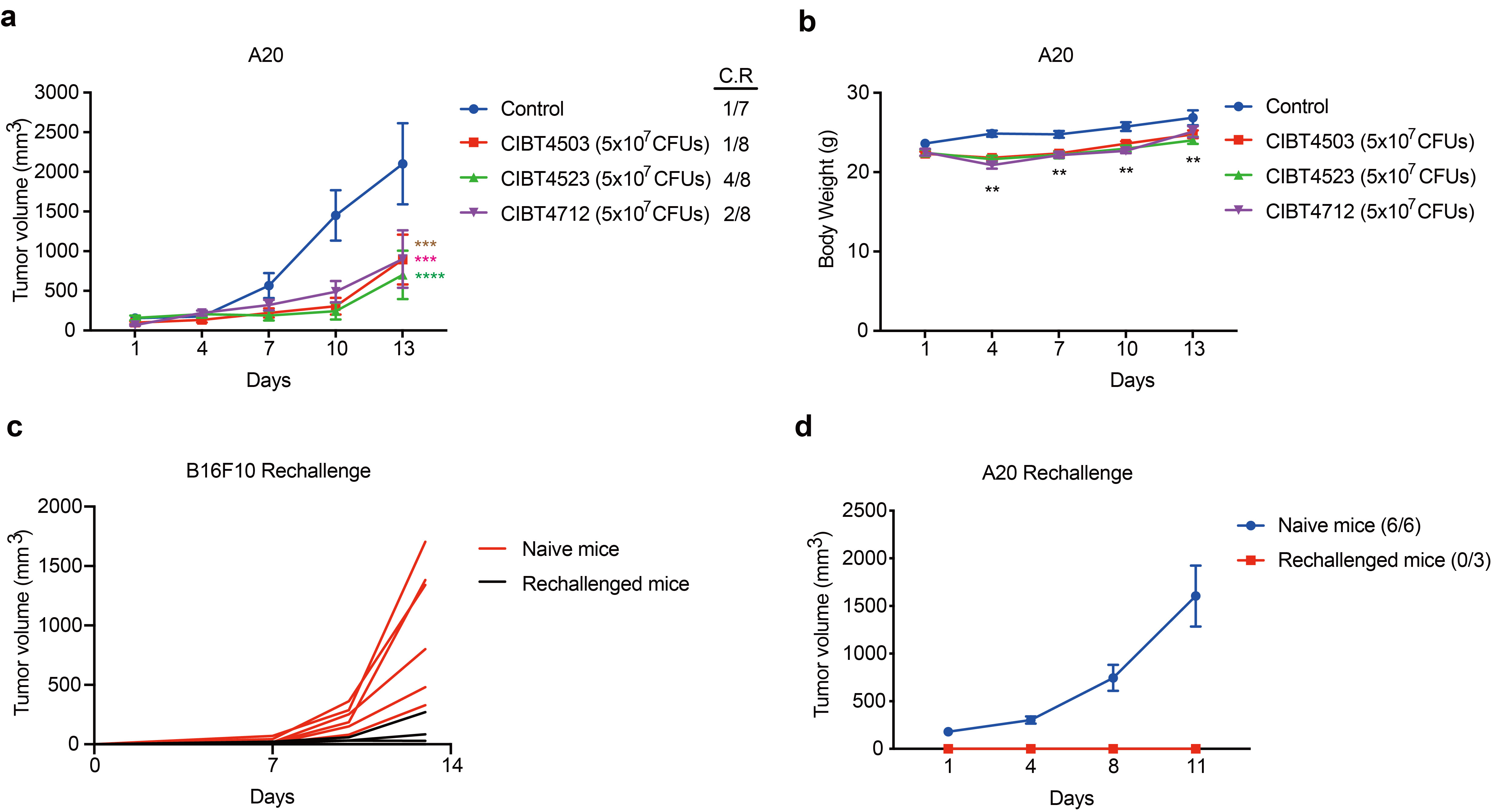


**Supplemental Figure 3.** Anti-tumor activity of the engineered strains in vivo. (a) A20 tumor-bearing mice were intratumorally treated with PBS (Control), CIBT4523 or CIBT4712 at days 1, 4, and 7, tumor growth (a) and body weight (b) were shown. (c) Tumor growth of each mouse in Figure 4g. (D) Three cured mice in (a) were kept tumor-free for two months, these mice and naïve mice were subcutaneously injected with A20 cells (5×10^5^) in the contralateral flank. The number of A20 tumor-bearing mice and total mice were shown. Data was expressed as Mean and S.E.M. Two-way ANOVA. ^**^*P* < 0.01.

**Supplementary Figure 4**. Cyclic-di-AMP abundance from tumor homogenates. The B16F10 (1 × 10^5^) cells were subcutaneously injected into C57BL/6 mice. When the tumors reached a volume of 300–500 mm^3^, the mice were randomized into different groups (n=6) and intraperitoneally injected with 60 µL of ATC (200 µg/mL), and 4 h later, 100 µL of CIBT4523 (1 × 108 CFUs), CIBT4503 (1 × 108 CFUs), or vehicle (PBS) was administered. After 24 h, the mice were euthanized, and the tumors were removed via dissection. CDA was measured by LC-MS/MS system.


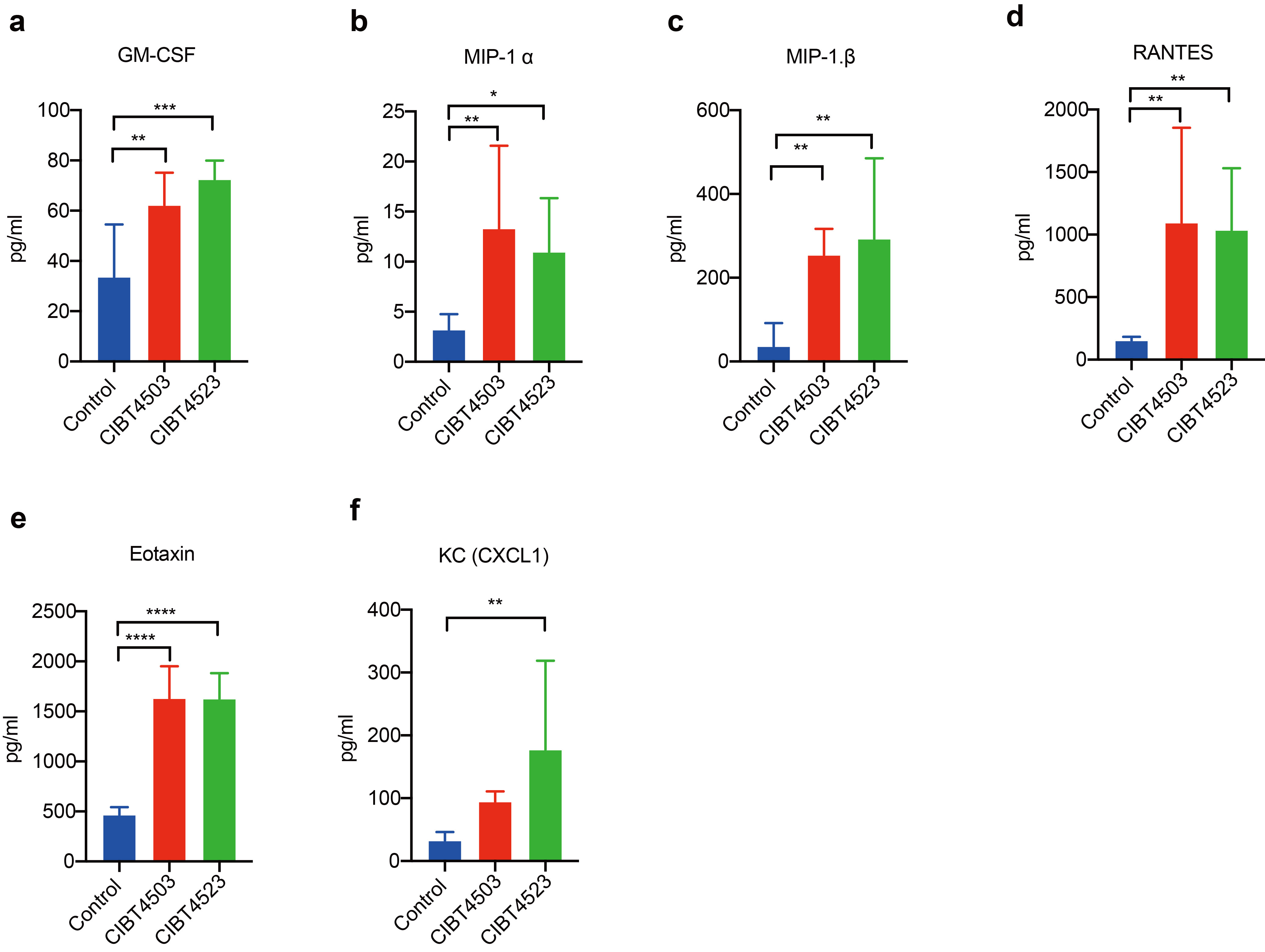


**Supplemental Figure 5. (a-f)** Concentration of cytokines in three groups, refer to Figure 6. ^*^*P* < 0.05, ^**^*P* < 0.01, ^***^*P* < 0.001, ^****^*P* < 0.0001, One-way ANOVA with Tukey’s multiple comparisons tests.

**References**

1 Song, X., Huang, H., Xiong, Z., Ai, L. & Yang, S. CRISPR-Cas9(D10A) Nickase-Assisted Genome Editing in Lactobacillus casei. *Appl Environ Microbiol* **83**, e01259-01217, doi:10.1128/AEM.01259-17 (2017).

2 Jiang, Y. *et al.* Multigene editing in the Escherichia coli genome via the CRISPR-Cas9 system. *Appl Environ Microbiol* **81**, 2506-2514, doi:10.1128/aem.04023-14 (2015).

3 Datsenko, K. A. & Wanner, B. L. One-step inactivation of chromosomal genes in Escherichia coli K-12 using PCR products. *Proc Natl Acad Sci U S A* **97**, 6640-6645, doi:10.1073/pnas.120163297 (2000).

4 Rennig, M. *et al.* TARSyn: Tunable Antibiotic Resistance Devices Enabling Bacterial Synthetic Evolution and Protein Production. *ACS Synth Biol* **7**, 432-442, doi:10.1021/acssynbio.7b00200 (2018).

5 Rennig, M. *et al.* Industrializing a Bacterial Strain for l-Serine Production through Translation Initiation Optimization. *ACS Synth Biol* **8**, 2347-2358, doi:10.1021/acssynbio.9b00169 (2019).

6 Gibson, D. G. *et al.* Enzymatic assembly of DNA molecules up to several hundred kilobases. *Nat Methods* **6**, 343-345, doi:10.1038/nmeth.1318 (2009).
